# Transcriptional atlas analysis from multiple tissues reveals the expression specificity patterns in beef cattle

**DOI:** 10.1186/s12915-022-01269-4

**Published:** 2022-03-29

**Authors:** Tianliu Zhang, Tianzhen Wang, Qunhao Niu, Lei Xu, Yan Chen, Xue Gao, Huijiang Gao, Lupei Zhang, George E. Liu, Junya Li, Lingyang Xu

**Affiliations:** 1 Institute of Animal Science, Chinese Academy of Agricultural Sciences, Beijing, 100193 People’s Republic of China; 2 Animal Genomics and Improvement Laboratory, United States Department of Agriculture, Agricultural Research Service, Beltsville, Maryland 20705 USA

**Keywords:** Gene expression, Housekeeping genes, Co-expression network, Tissue-specific genes, Differentially expressed genes, Beef cattle

## Abstract

**Background:**

A comprehensive analysis of gene expression profiling across tissues can provide necessary information for an in-depth understanding of their biological functions. We performed a large-scale gene expression analysis and generated a high-resolution atlas of the transcriptome in beef cattle.

**Results:**

Our transcriptome atlas was generated from 135 bovine tissues in adult beef cattle, covering 51 tissue types of major organ systems (e.g., muscular system, digestive system, immune system, reproductive system). Approximately 94.76% of sequencing reads were successfully mapped to the reference genome assembly ARS-UCD1.2. We detected a total of 60,488 transcripts, and 32% of them were not reported before. We identified 2654 housekeeping genes (HKGs) and 477 tissue-specific genes (TSGs) across tissues. Using weighted gene co-expression network analysis, we obtained 24 modules with 237 hub genes (HUBGs). Functional enrichment analysis showed that HKGs mainly maintain the basic biological activities of cells, while TSGs were involved in tissue differentiation and specific physiological processes. HKGs in bovine tissues were more conserved in terms of expression pattern as compared to TSGs and HUBGs among multiple species. Finally, we obtained a subset of tissue-specific differentially expressed genes (DEGs) between beef and dairy cattle and several functional pathways, which may be involved in production and health traits.

**Conclusions:**

We generated a large-scale gene expression atlas across the major tissues in beef cattle, providing valuable information for enhancing genome assembly and annotation. HKGs, TSGs, and HUBGs further contribute to better understanding the biology and evolution of multiple tissues in cattle. DEGs between beef and dairy cattle also fill in the knowledge gaps about differential transcriptome regulation of bovine tissues underlying economically important traits.

**Supplementary Information:**

The online version contains supplementary material available at 10.1186/s12915-022-01269-4.

## Background

As one of the most important farm animals, cattle is a significant source of milk, meat, and hides and contributes to human diets and agricultural economics [1]. Advanced technologies have promoted the genetic improvement of both beef and dairy cattle by effectively utilizing genomic information [2]. A better understanding of the functional components is the main strategy to explore the genetic basis of important traits and improve the accuracy of genomic prediction in farm animals [3–5]. Many studies have been carried out to explore gene expression atlas and genome annotations in various mammals, especially for humans, mice, and other model animals [6–9]. For example, the Function Annotation of the Mammalian Genome (FANTOM) Consortium [10] and Encyclopedia of DNA Elements (ENCODE) project [11] were proposed to help elucidate various human disease genes and identify functional elements in the human genome sequence. Several International Consortium projects, including Genotype-Tissue Expression (GTEx) [12] and International Human Epigenome Consortium (IHEC) [13], were launched to identify the relationship between genetic variation and gene expression in human tissues and to interpret epigenetic control of cell states relevant for human health and disease. Moreover, to create a comprehensive framework for biological system analysis on farm animals and improve functional annotation of animal genomes, the FAANG (Functional Annotation of ANimal Genomes) Consortium was organized to generate a comprehensive genome-wide data sets on RNA expression, DNA methylation, and chromatin modification, as well as chromatin accessibility and interactions [14]. Recently, the transcriptome analyses from long-read sequencing technology (e.g., Pacific Biosciences Iso-Seq, Oxford Nanopore technology) can help to improve the annotation of the transcriptome in farm animals [15, 16].

A high-quality reference genome with enhanced assembly accuracy can provide precise genome sequence information to improve the gene annotations and other genomic features [17]. In cattle, the latest reference genome ARS-UCD1.2 increased the overall continuity of the genome by reducing gaps and inversions using long-read sequence assembly methods when compared to UMD3.1 [15]. Thus, it can improve the genome annotation by utilizing the representation of low abundance and tissue-specific transcripts based on available public and the newly sequenced Iso-Seq datasets [17]. Previous studies presented a comprehensive transcriptome survey based on ninety-five samples from three growth stages and one cell line [18] and identified a subset of functional enrichment of enhancer regions [19]. These effects promoted the establishment of a Bovine Genome Database (BGD) [20] and facilitated the deposition, curation, annotation, and integrated analysis of genomic data for global research communities [21, 22].

Investigating the expression pattern of genes in different tissues can help elucidate an organism’s evolutionary mechanisms and biological functions [23]. Especially, different expression patterns across tissues could offer valuable insights into understanding the genetic basis underlying the breed formation in cattle (e.g., beef and dairy cattle). Previous transcriptome studies have reported the gene expression atlas for multiple domestic animals, including cattle, pigs, goats, sheep, and water buffalo [18, 24–27]. Differentially expressed genes (DEGs) or core driver genes were identified as potential candidates, which were involved in important functions, including growth and development [28, 29], meat quality [30, 31], wool follicle [32, 33], and disease-resistance [34, 35]. Meanwhile, organ and tissue-specific gene expression patterns were also studied in farm animals [18, 36–38], and these analyses further facilitated the elucidation of the relationship between gene expression, tissue, and organ. However, most bovine studies were carried out in dairy cattle [39, 40], while a few studies were reported in beef cattle, and many of them with a limited number of tissue types and outdated sequencing platforms. Therefore, functional annotation based on improved assembly and comparative analysis of tissue-specific expression patterns using a large scale of gene expression atlas are urgently needed in beef cattle.

In this study, we performed a comprehensive transcriptome analysis on 51 types of bovine tissues (heart, liver, spleen, brain, muscle, adipose, cartilage, gland, etc.) from adult beef cattle. We investigated housekeeping genes (HKGs), tissue-specific genes (TSGs), and co-expression hub genes (HUBGs), and analyzed their expression regulation patterns. Moreover, we explored candidate DEGs and their functional pathways related to important traits between beef and dairy cattle. Our study provides a valuable resource to improve the genome annotation of the current reference genome for cattle, and these findings can further contribute to a better understanding of the genetic basis underlying the complex traits in farm animals during breed formation.

## Results

### Summary statistics of sequencing dataset

**Fig. 1:**
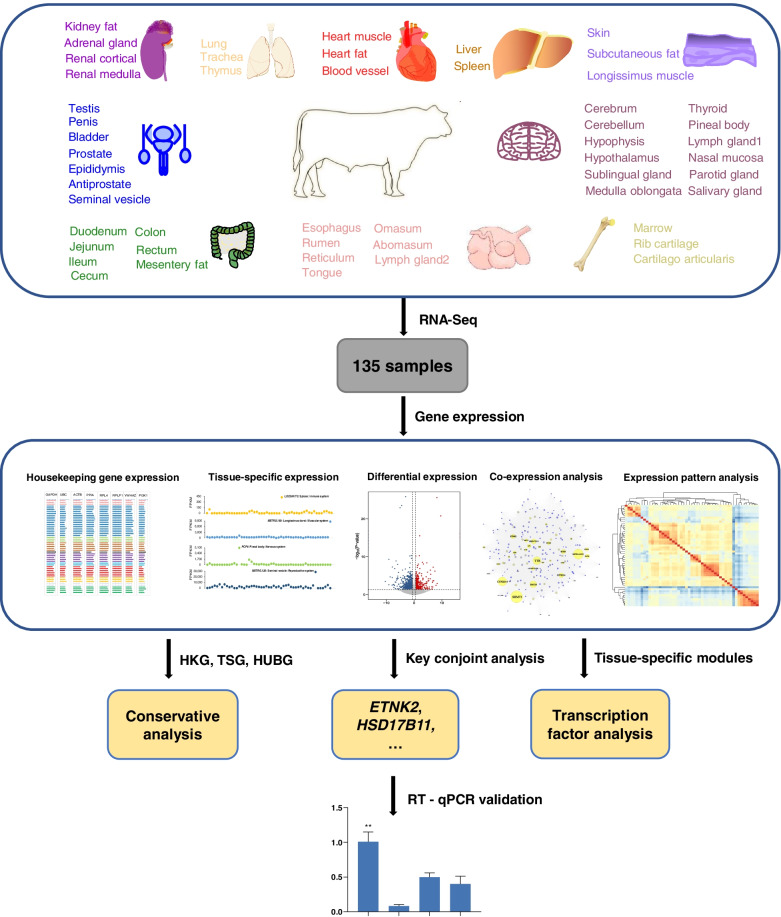
Global framework of the current study. We used 51 tissues from male Chinese Simmental beef cattle to study the expression specificity patterns through multifaceted analyses (tissue-specific expression, differentially expressed gene analysis, co-expression analysis, expression pattern analysis, housekeeping gene expression, etc.). Then, we performed conservation analysis and RT-qPCR validation for several identified candidate genes. The panel at the top shows the tissue samples. Tissues belonging to the same organ system are labeled with the same color

We generated a large-scale gene expression profile covering 51 types of tissues from three adult male Chinese Simmental beef cattle. The tissue types for our dataset were illustrated in Fig. 1, which represents major organ systems (i.e., circulatory system, digestive system, endocrine system, immune system, muscular system, nervous system, reproductive system, respiratory system, skeletal system, and urinary system). A detailed list of tissues and corresponding organ systems was presented in (Additional file 1: Table S1). We adopted a uniform library building strategy (in Methods) for tissue samples to reduce the influence of library batch effect on expression level. Our study generated approximately 7 billion raw paired-end reads (~1151 GB), with an average of 56 million reads per sample. After strict quality control (see the “Methods” section), a total of 1066 GB of high-quality data was kept for subsequent analysis. Based on the latest released reference genome (ARS-UCD1.2), the high-quality sequencing data were mapped using the HISAT2 software with the default setting [41]. The average reads mapping rate was ~94.76% (ranging from 86.97 to 96.90%) among 135 bovine tissue samples. Detailed mapping information and summary statistics for each sample were shown in (Additional file 1: Table S2).

### Annotation of the bovine genes using transcriptome atlas

The genome annotation with improved assembly can help us understand genes involved in biological pathways and the regulatory relationship of expression processes [3]. To further assess the abundance of transcripts across tissues, we first estimated the number of transcripts (requiring FPKM > 1) in each sample (ranging from 11,540 to 30,028). Of these, around 65.4% to 71.9% were annotated as protein-coding genes (*n* = 8299 to 19,649) (Additional file 1: Table S3). The count variations of transcripts and annotated protein-coding genes across tissues may be related to tissue-specific gene expression patterns.

To identify novel transcripts, we merged all assembled transcripts across samples and compared the merged assembled transcript with bovine reference annotation (ARS-UCD1.2) using the GFFCOMPARE program. We then determined the counts of completely assembled transcripts and partially matched the reference annotated transcripts. Our results showed that the total number of query mRNA transcripts was 185,955 (165,276 multi-exon transcripts), while the number of matched reference mRNA transcripts was 82,206 (73,049 multi-exon transcripts) (Additional file 1: Table S4). In addition, we explored the match patterns between the assembled transcripts and reference annotated transcripts based on the “class code” as described by [42]. Our results revealed that the proportion of transcripts matching precisely with the introns (codes as “=”) from the two annotation files was up to 44.19%, and the number of potential novel isoforms (coded as “j”) was 78,032 (41.96%). However, the predicted transcript within the reference transcript (codes as “c”) was 0 (Additional file 1: Table S5). Our findings also revealed different matching patterns of transcripts in diverse tissues compared to reference genome annotation. Due to the incomplete and complexity of transcriptome annotation in domestic animals [17, 43], further experimental validations were required to confirm whether they are novel transcripts or assembly artifacts [42].

### Gene expression profile across tissues

**Fig. 2:**
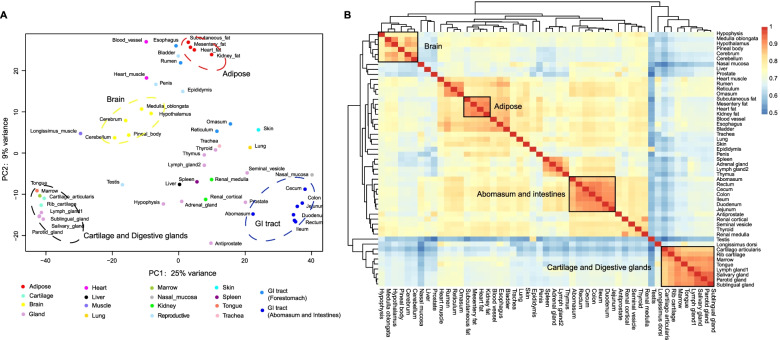
Gene expression profile among 51 tissue types. **a** Principal component analysis for all tissue types based on corrected expression data through log_2_ (FPKM+1). Tissues are colored according to organ systems as the same as in Fig. 1. **b** Unbiased hierarchical clustering heat map based on Pearson’s correlation coefficient for all genes. Color intensity indicates the correlation between tissues, red indicates high correlation (1), and blue indicates low correlation (0.5)

To explore the diversity and biological logical clustering across bovine tissues, we retained 12,588 genes expressed in at least two sample replicates of each tissue and converted FPKM values using log-transformation (log_2_ (FPKM+1)). Using principal component analysis (PCA), we observed that tissues with similar physiological functions were more likely to cluster together, which was consistent with the previous analysis of the BRENDA database [44] (Fig. 2a). For instance, medulla oblongata, hypothalamus, pineal body, cerebrum, and cerebellum tissues, belonging to the central nervous systems were clustered together. For adipose-related tissues, we observed that mesentery fat, heart fat, kidney fat, and subcutaneous fat were grouped together. Moreover, we observed that the hierarchical clustering and correlation between the bovine tissues agreed with the result of PCA analysis (e.g., the digestive system was composed of two branches, the front stomach, abomasum, and intestine) (Fig. 2b). These results also implied that the changes in gene expression profiles among various tissues might be involved with tissue differentiation.

### Housekeeping gene expression patterns across bovine tissues

We defined HKGs as constitutively expressed genes, which were expressed in all tissues to maintain basic necessary biological processes and cell functions (e.g., cellular transport and cell cycle) [45]. Totally, we identified 2654 genes constitutively expressed across 51 tissues. Additionally, we quantified the expression variability of these genes between tissues using the coefficient of variation (CV). As expected, we observed that CV values of HKGs were smaller when compared with the full gene set, which may indicate the expression of HKGs tends to be constant among tissues (Additional file 2: Fig. S1).

**Fig. 3:**
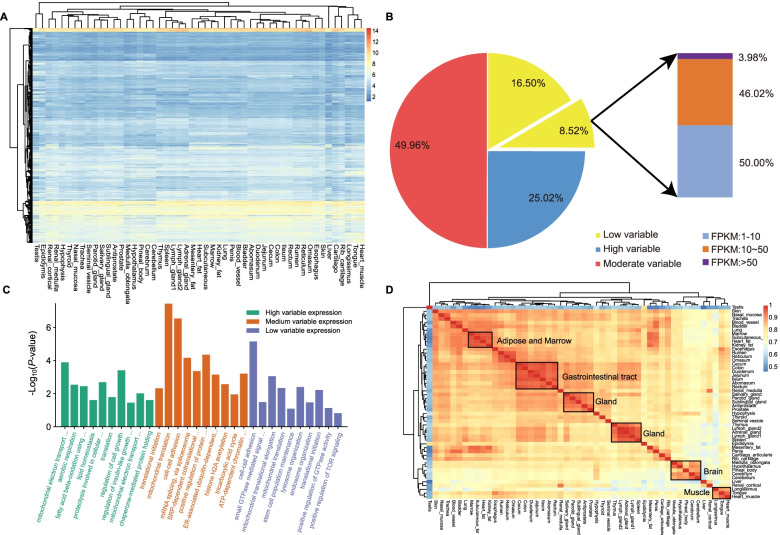
The expression pattern and hierarchical clustering of 2654 HKGs across 51 bovine tissues. **a** Clustering of expression patterns of housekeeping genes. Color intensity represents expression level estimated through log_10_ normalized FPKM. Red indicates high expression and blue indicates low expression. **b** The HKGs are variably expressed and only 8.52% are constantly expressed HKGs. Among those constant HK genes, only 3.98% are highly expressed with FPKM larger than 50. **c** Functional annotation of low variable expression, medium variable expression, and high variable expression of HKGs. **d** Hierarchical clustering heatmap based on Pearson’s correlation coefficient for HKGs. The red color represents high correlation and the blue color represents low correlation

On the other side, we did observe that the expression levels of HKGs varied among tissues (Fig. 3a). Based on the CV, we further classified HKGs into three groups with low, medium, and high expression variability using thresholds of 0.35 (first quartile) and 0.66 (third quartile) (Fig. 3b). Gene Ontology (GO) enrichment analysis showed that genes with low and medium expression variability were mainly involved in maintaining the basic biological activities of organisms (e.g., cell-cell adhesion (GO:0098609), translational initiation (GO:0006413) and mitochondrial translation (GO:0032543)). In contrast, genes with high expression variations were involved in energy metabolism (e.g., mitochondrial electron transport, ubiquinol to cytochrome c (GO:0006122), aerobic respiration (GO:0009060)) (Fig. 3c).

Out of 223 HKGs with low expression variability, we filtered out 96.02% of them with low expression (1 < FPKM ≤ 10) or medium expression (10 < FPKM ≤ 50). Finally, we obtained 32 HKGs with high expression levels and low expression variability across tissues, which may serve as valuable experimental controls in gene expression experiments (Additional file 3:Table S6, Additional file 4: Fig. S2). Remarkably, we found that several other commonly used or commercially available HKGs were not presented in the list of 32 HKGs (Additional file 4: Fig. S3). For example, a commonly used HKG *ACTB* [46] was actually not constantly expressed in most of the bovine tissues.

To understand the potential contribution of 2654 HKGs identified from bovine tissues, we performed a correlation analysis between pairwise tissues using Pearson correlation (Fig. 3d). Our results showed that tissues with similar physiological functions have higher correlation coefficients and these tissues were mainly clustered together, and these results were in line with results obtained from the full gene list (Fig. 2b). Interestingly, the testis tissue was grouped into a single branch, and this finding may indicate different expression patterns and functional specificity of HKGs in bovine testis compared to other tissues.

### Tissue-specific expression patterns in bovine organ systems

**Fig. 4:**
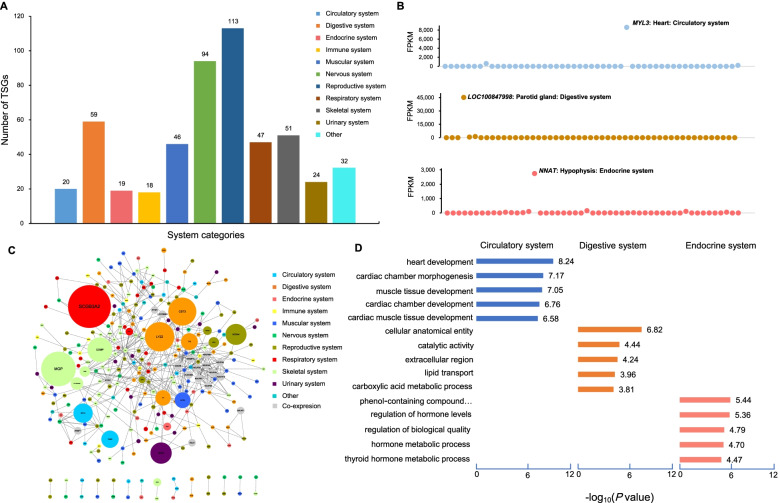
Tissue-specific expression patterns between system categories. **a** Distribution of the number of tissue-specific genes in all system categories. **b** Examples of TSG in the circulatory system (*MYL3*), the digestive system (*LOC100847998*) and the endocrine system (*NNAT*). The *x*-axis represents tissue labeled with the same colors as in Fig. 1 and the *y*-axis is the FPKM value. **c** Network topology analysis of 477 TSGs based on the String database. Each node represents a tissue-specific protein-coding gene. The size of the node indicates the level of expression of tissue-specific protein-coding genes. The node colors represent ten system categories, and the gray means that the gene comes from the STRING database. The edges represent the co-expression relationship between tissue-specific protein-coding genes. **d** Functional annotation and enrichment distribution of tissue-specific gene sets in the system. The *x*-axis represents -log_10_ (*P* value) and the *y*-axis represents GO term

Using stringent criteria as described by a previous study [47], we identified 477 TSG, together with 10,814 tissue-specific transcripts (TSTs). Among tissues, we detected the largest (*n* = 51) TSGs in the prostate (Additional file 5: Table S7), followed by 46 TSGs in longissimus dorsi. The number of TSGs in other tissues ranges from 0 to 39. We partitioned the 51 tissues into ten organ system categories to estimate important lineage-related genes during tissue development. Notably, we observed that the reproductive system had the most tissue-specific genes (i.e., 113) and the number of TSGs in all other organ systems ranges from 18 to 94 (Fig. 4a). We obtained a serial of highly expressed TSGs in each organ systems category. For instance, *MYL3* was the gene with the highest expression in the circulatory system, while *LOC100847998* and *NNAT* showed the highest expression in the digestive and endocrine systems, respectively (Fig. 4b). Tissue-specific expression analyses of other organ system categories were shown in (Additional file 6: Fig. S4). Moreover, we performed the protein interaction network analysis of TSGs in different organ systems based on the STRING database [48]. Our results showed that TSGs exhibited a co-expression regulation pattern and the top TSGs displayed higher expression levels when compared with other genes in the organ system (Fig. 4c). The functional annotation and pathway enrichment analyses of TSGs using KOBAS (v2.0) for different organ systems further support the known biological functions of tissues, which agreed with many previous studies [49–51] (Fig. 4d, Additional file 6: Fig. S5). For instance, the TSGs related to the circulatory system were significantly enriched in heart development (corrected *P* value = 4.23e−06) and cardiac chamber development (corrected *P* value = 6.80e−08), the digestive system for catalytic activity (corrected *P* value = 1.24e−02) and lipid transport (corrected *P* value = 1.59e−02), the endocrine system for regulation of hormone levels (corrected *P* value = 1.63e−03) and hormone metabolic process (corrected *P* value = 3.69e−03) (Additional file 7: Table S8).

#### The digestive system

The specialized digestive system consisting of the digestive tract and digestive glands enables ruminant animals like cattle to efficiently digest roughage. As it was described in Fig. 2b, the bovine digestive tract was divided into anterior (esophagus, rumen, reticulum, omasum) and posterior (abomasum, duodenum, jejunum, ileum, cecum, colon, rectum) clusters based on tissue-specific expression patterns. We identified 5, 10, and 5 gene set specific to the anterior, posterior of the digestive tract and digestive glands, respectively (Additional file 5:Table S7). We found that the functional annotation of digestive system tissue-specific genes also corresponds to its known tissue-related biology. The digestive system anterior TSGs (like *FAM83D* and *GALNT6*) were mainly involved in the nutrients catabolic process and microtubule binding. In addition, TSGs (e.g., *CYP4V2*) in liver tissue were involved in the metabolism of nutrients (i.e., lipid transport, fatty acid oxidation, and amino acid metabolism).

#### The central nervous system

Remarkably, we identified a total of 48 TSGs (ranging from 3 to 20) in brain tissues (the central system) for each tissue. The identified expression pattern suggested that the pineal gland was separated from other brain tissues (Additional file 8: Fig. S6), indicating the diverse functions among the brain tissues. Notably, the TSG expression levels were also variable among brain tissues, confirming their tissue-specific expression patterns. For instance, several TSGs were identified in the cerebellum (*CBLN3*), cerebrum (*NSMF*, *VSTM2L*, and *ENC1*), hypothalamus (*Map2k7*), and pineal gland (i.e., *ROM1*, *UNC119*, *GNB3*, and *GNG13*).

### Co-expression gene network analysis and HUBGs

**Fig. 5:**
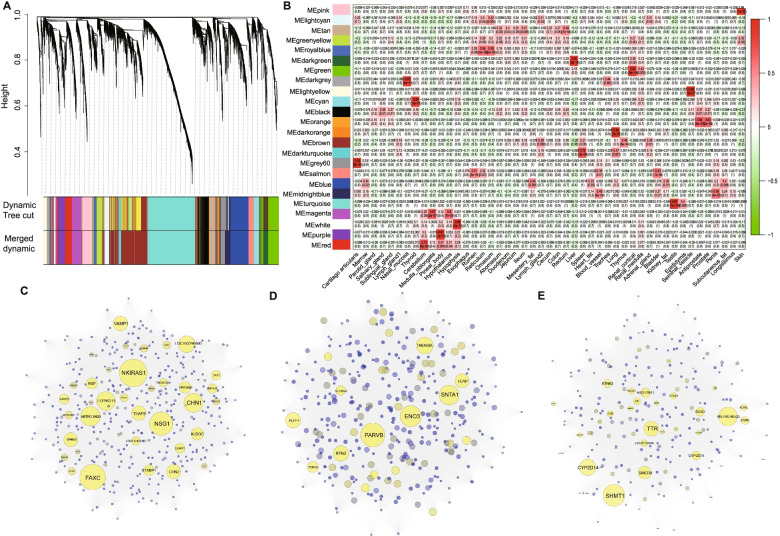
Clustered network graph of the transcriptome in bovine tissues. **a** Functional modules are represented in different colors. Each major branch represents a color-coded module that contains a group of highly connected genes. **b** Heatmap between 24 modules and 47 tissues. Boxes display Pearson correlation coefficients and their associated P values. Red indicates that the given tissue has a strong positive correlation relative to all other tissues. Green indicates that the given tissue has a strong negative correlation relative to other tissues. **c**, **d**, and **e** represents the cerebellum-red module, the muscle-pink module, and the liver-dark green module, respectively. Hub genes were marked with yellow. The size of each node represents the within module connectivity of the node to adjacent genes

We constructed networks based on weighted gene co-expression network analysis (WGCNA) to explore the biological relationships and potential functions of core driver genes across tissues. In the current study, a total of 8357 filtered genes (FPKM > 1) were used for subsequent analyses. Gene-based expression matrix clusters were generated to illustrate the relationships between samples. To avoid the influence of low-quality samples, we only kept the samples in cluster 1 with 47 samples for co-expression network analysis (Additional file 8: Fig. S7). A soft thresholding value *β* was of 4 selected when the *R*^2^ = 0.85, based on the criteria outlined by Zhang et al. [52]. (Additional file 8: Fig. S8). The estimated k was highly correlated with p(k) (*R*^2 ^= 0.82), indicating that the selected β value can effectively establish a scale-free network (Additional file 8: Fig. S9). We calculated the dissimilarity of the genes based on the converted topological matrix and generated hierarchical clustering (Fig. 5a). Using co-expression network analysis, we obtained 24 modules based on bovine transcript atlas (the number of genes for each module ranged from 68 to 1710) (Additional file 9: Table S9). To obtain tissue-specific modules, we assessed the association between 24 modules and 47 tissues. Under the criteria of the correlation coefficient (*r* > 0.65) and *P* value (*P* < 1.0e−4), we identified 15 tissue-specific modules (Additional file 9: Table S10). For instance, the light yellow module and the pink module have high correlation coefficients with seminal vesicle glands (*r* = 0.96, *P* value = 2.00e−27) and longissimus dorsi (*r* = 0.96, *P* value = 1.00e−25), respectively (Fig. 5b). The turquoise module with the largest gene count (1710) was closely related to testis (*r* = 0.87, *P* value = 1.00e−15). We also observed that the functional annotations of genes in tissue-specific modules correspond to tissue function. For instance, genes within the red module (cerebellum) and magenta modules (medulla oblongata) were involved in the morphological development of neurons and axons. The dark green module corresponds to the liver, and the genes with this module were found to be enriched in small molecule metabolism and the oxidation-reduction process. The pink module was related to muscle and involved in energy metabolism processes and cardiac muscle contraction (Additional file 9: Table S11). We then calculated the network connectivity (kTotal) and within module connectivity (kWithin) for each gene (Additional file 9: Table S12). The genes in the module with the highest connectivity (top 5%) were selected as HUBGs. We finally identified a total of 237 HUBGs in 47 bovine tissues (Additional file 9: Table S13). We observed HUBGs of tissue-specific modules in the network diagram (e.g., cerebellum-red module, muscle-pink module, liver-dark green module) (Fig. 5c–e). Notably, we also found that a subset of HUBGs in the module also showed tissue-specific expression. For instance, seven genes (*GATA2*, *PLA2G7*, *SLCO2A1*, *CCL2*, *RNF113A*, *SMARCA1*, and *FAM84A*) in the light yellow module (seminal vesicle gland), and eighteen genes (*BIN1*, *DUSP15*, *ENO3*, *FEM1A*, *LOC616200*, *GYS1*, *GYS1*, *KEAP1*, *PARVB*, *PLPP7*, *PPP1R1A*, *RTN2*, *SLC16A5*, *SNTA1*, *TCAP*, *TMEM38A*, and *ZNF358*) in the pink module (longissimus dorsi).

To explore master regulators of genes in co-expressed networks, we performed transcription factor (TF) analysis on 237 genes in the dark green module and 355 genes in the pink module. For the dark green module, we identified 11 TFs including HNF4G (normalized enrichment score (NES) = 6.296), SREBF1 (NES=6.290), HNF4A (NES=5.208), SREBF2 (NES=4.169), HDAC2 (NES=3.843), EP300 (NES=3.639), CEBPB (NES=3.581), ZEB1 (NES=3.486), SP4 (NES=3.434), GLIS1 (NES=3.402), and FOXA2 (NES=3.12) (Additional file 10: Fig. S10). For the pink module, 5 TFs, including HCFC1 (NES=3.930), PBX3 (NES=3.538), NFKB1 (NES=3.342), JUND (NES=3.224), and KLF3 (NES=3.126), were detected in our analyses (Additional file 11: Table S14).

### Conservation of HKGs, TSGs, and HUBGs across species

To evaluate the extent of conservation for HKGs, TSGs, and HUBGs, we assessed the number of orthologs of bovine genes in multiple eukaryotic species to investigate functional conservation across species [53] (Additional file 11: Table S15). Using NCBI HomoloGene, we observed that HKGs, TSGs, and HUBGs were more likely to be orthologs in other species based on Student’s *t*-test (*P* = 1.49e−03, *P* = 3.40e−04 and *P* = 3.07e−04, respectively). However, few orthologs genes were identified for HKGs, TSGs, and HUBGs as the evolutionary distance between bovine and these eight species increase. Compared to vertebrates, the ortholog ratios of HKGs, TSGs, and HUBGs in invertebrates (i.e., worm, fly, and yeasts) were lower. Moreover, we observed that the ortholog proportion of HKGs was higher than that of TSGs. Our analysis showed that most HKGs in cattle were conserved, which were mainly involved in fundamental cell survival compared with TSGs contributing to tissue-specific differentiation [54]. To determine whether the HKG expression pattern was conserved across species, we estimated the normalization average expression level and CV of bovine HKGs orthologs and compared with those of humans, mice, rats, and dogs as described by [47]. Our analyses showed that the expression level of bovine HKG was stable with the smallest CV value, and the *P* value was 1.49e−3 cross species using Student’s *t* test (Additional file 11: Table S16). These findings suggested that the expression patterns of HKGs were conserved among them. Our results also confirmed that the HKGs were more ancient than TSGs and HUBGs with tissues specific expression patterns [45].

### Comparative analysis of differentially expressed genes expression patterns between cattle breed (beef vs. dairy cattle)

**Fig. 6:**
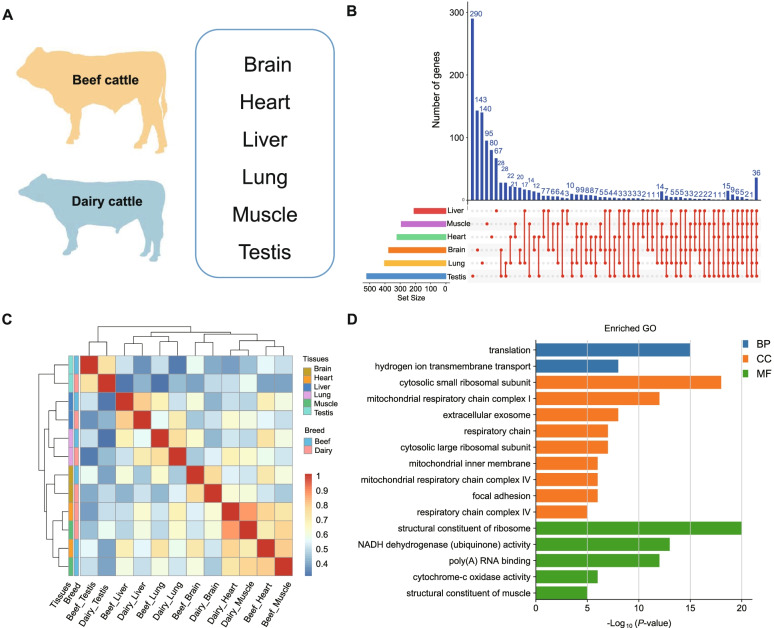
Different gene expression patterns between beef and dairy. **a** Overview of tissues collected from adult beef and dairy cattle for RNA-seq. **b** Venn diagram shows the shared and unique differentially expressed genes among heart, muscle, liver, lung, brain, and testis tissues between beef and dairy cattle. **c.** Symmetric heatmap generated based on the Spearman correlation coefficients of all differentially expressed genes in all paired wise tissues. **d** GO function annotation of DEGs in muscle tissue. BP represents biological process, CC represents cellular component, and MF represents molecular function

The diverse phenotypes between beef and dairy cattle imply the potential difference of genetic basis under selection (meat vs. milk purpose) during the breed formation. Here, we evaluated transcriptome changes using RNA-seq on the six primary bovine tissues (i.e., brain, heart, liver, lung, muscle, and testis) of beef and dairy cattle [4] and investigated the differentially expressed genes and gene expression profiles among tissue categories (Fig. 6a). We observed that the number of DEGs among tissues ranges from 209 in lung tissue (109 upregulated and 100 downregulated genes) to 521 in testis tissue (356 upregulated and 165 downregulated genes). For muscle tissue, we found 403 DGEs, including 214 up-regulated and 189 down-regulated genes (Additional file 11: Table S17). To better understand tissue-specific DEGs, we performed comparative analyses of DEGs among the six tissues (Fig. 6b). Our results showed that 36 DEGs were shared by the six tissues. In addition, the testis has the most tissue-specific DEGs (290). To further explore the biological expression patterns of tissue-specific DEGs, we calculated the correlation coefficient and assessed the clusters pattern between tissues. Remarkably, our results showed that samples from the same tissues of beef and dairy cattle were clustered together based on the differential gene expression profile (Fig. 6c). We found that samples from heart and muscle tissues were clustered together in our analyses, and this may imply muscle tissue may have similar expression patterns within heart tissues for both beef and dairy cattle.

To assess the functional contribution of DEGs for important traits in cattle, we performed functional enrichment analysis using DAVID Bioinformatics Resources 6.8 [55]. DEGs identified across tissues were enriched in molecular function (MF) GO terms including a structural constituent of ribosome (GO:0003735), NADH dehydrogenase (ubiquinone) activity (GO:0008137); biological process (BP) terms including translation (GO:0006412), hydrogen ion transmembrane transport (GO:1902600), and cellular component (CC) terms includes respiratory chain (GO:0070469), cytosolic small/large ribosomal subunit (GO:0022627, GO:0022625) (Additional file 11:Table S18).

DEGs annotated in muscle may probably indicate their specific functions contributing to production traits in beef cattle. Remarkably, we identified several candidate DEGs (e.g., upregulated genes like *TPM1*, *TPM3*, *COX4I1*, *COX5A*, and *NDUFA4*, with approximately 3- to 6-fold expression increases in beef cattle) related to muscle differentiation and development, including the structural constituent of muscle (GO:0008307) and cytochrome-c oxidase activity (GO:0004129) (Fig. 6d). Several genes with oxidative phosphorylation (bta00190) were highly expressed in the liver of beef cattle compared to dairy cattle, with approximately 3- to 32-fold expression increases for *SDHA*, cytochrome C oxidase members (*COX7A2*, *COX7B*), and NADH dehydrogenase family members (*NDUFAB1*, *NDUFB9*, and *NDUFC1*). Moreover, several DEGs identified in the liver were enriched in pathways related to digestion and metabolism diseases, including primary bile acid biosynthesis (bta00120), fatty acid metabolism (bta01212), and non-alcoholic fatty liver disease (NAFLD) (bta04932) (Additional file 12: Fig. S11). Notably, we obtained 67 and 323 DEGs which were HKGs and TSGs, respectively. Functional enrichment results showed that TSGs were enriched in metabolic pathways, including fructose and mannose metabolism, galactose metabolism, and pentose phosphate pathway, while HKGs were enriched in ribosome and proteasome.

### Validating expression profiles with known tissue-specific genes across bovine tissues

To verify the expression accuracy of TSGs identified from RNA-seq, we performed RT-qPCR on four tissues (muscle, liver, brain, and abomasum) from the same beef cattle. Twelve tissue-specific genes were randomly selected, including two muscle-specific genes (*ALDOA*, *HSPB7*), four liver-specific genes (*CYP4V2*, *ETNK2*, *HSD17B11*, *SC5D*), three brain-specific genes (*ENC1*, *NSMF*, *VSTM2L*), and three abomasum-specific genes (*CA9*, *KRT18*, *MUC5AC*). RT-qPCR primers using the Primer Premier 5 for all genes were presented in (Additional file 13:Table S19).

Our results revealed that the expression of *ALDOA* and *HSPB7* in muscle was significantly higher than the other three tissues (Additional file 14: Fig. S12). Our results also showed that *CYP4V2*, *ETNK2*, *HSD17B11*, and *SC5D* were highly expressed in liver tissue, *ENC1*, *NSMF*, *VSTM2L*, were highly expressed in brain tissue, and *CA9*, *KRT18*, and *MUC5AC* genes were highly expressed in abomasum tissue. Moreover, we observed high concordances (*R*^2^ = 0.68~0.94) between RT-qPCR and RNA-seq (Additional file 14: Fig. S13). Therefore, our RT-qPCR results further confirmed tissue-specific expression of TSGs identified from RNA-seq.

## Discussion

### Bovine transcript atlas in beef cattle

A comprehensive survey of transcript abundance among tissues can provide valuable insights into elucidating biology function and regulation mechanisms of genetic variants that underlie complex traits [4, 56]. Previous studies using gene expression analysis suggested transcriptome changes from various tissues contribute to phenotypic diversity among species [57]. Gene expression differences underlying the important traits may also be associated with domestication and breed formation [58] (e.g., meat quality [30] and milk production [59]). However, transcriptome atlas analysis was limited by tissue types and sequencing platforms, which reduces the sensitivity to low-expressed transcripts. In mice and humans, more attention has been paid to investigate transcriptome changes from large-scale tissues for basic biological development at the whole genome level [60, 61]. For cattle, several studies have been conducted by focusing on gene expression changes among muscle [62], adipose [63], and rumen [64] tissues at different developmental stages or under specific conditions. Despite the first Bovine Gene Atlas had been reported using ninety-five samples with different tissue types and cell lines [18], this study was generated from digital gene expression tag sequences, which may fail to capture expression information from transcripts lacking DpnII sites. Subsequent studies based on tissue atlas have been reported on dairy cattle [4]; however, systematic analysis of tissue atlas for beef cattle was not fully addressed. In this study, we generated for the first time transcriptional atlas in beef cattle, which contains 135 samples with fifty-one tissue types representing ten organ systems (e.g., circulatory system, digestive system). By using a comprehensive survey of the transcriptome profiles and the comparative analysis of expression changes across multiple tissues, our results contributed to an in-depth understanding of the influence of gene expression variations from multiple tissues on phenotypic diversity.

Cattle have a complicated digestive system as compared to other mammals; thus, it was noted that some tissues (alimentary canal, including the rectum, cecum, colon, ileum, duodenum, jejunum, and abomasum) were tightly clustered, while other digestive tissues (esophagus, rumen, reticulum, omasum) were dispersed. This finding may be explained by the functional complexity and diversity of different tissues in the bovine gastrointestinal tract (GI tract). As for other bovine tissue types, we observed exocrine gland, endocrine gland, and deputy gonad were not well clustered, which may indicate their complex gene expression changes in different physiological functions.

### Constitutively expressed across tissues

Housekeeping genes were defined as those genes which were widely expressed across tissues, which can comprehensively represent the minimum set of genes required for the maintenance of basal cellular functions, and effectively be used as internal controls for experimental studies [65]. In the current study, we identified a total of 2654 housekeeping genes and 223 of them were constitutively expressed. In particular, 32 HKGs showing high express levels may be considered as feasible candidates for valuable experimental controls. In our study, the commonly used HKGs were expressed in all types of bovine tissues showing various expression levels, which was consistent with previous reports [66, 67]. We also obtained a list of HKGs involved in maintaining basic cell functions and energy metabolism (e.g., cell-cell adhesion, translational initiation, and aerobic respiration).

### Tissue-specific expression of diverse organ systems

Understanding the TSGs representing specific physiological processes can help enhance the understanding of the genetic and biological processes of complex traits [4]. We totally identified 477 TSGs from 51 types of tissues representing ten organ systems [47] (Additional file 5:Table S7). Functional enrichment analysis showed that TSGs mainly were related to the specific physiological functions of organ system categories. For the digestive system, *FAM83D* was overexpressed in the front stomach tissue, and this gene was related to regulating the regrowth of microtubules in a cell and serves as a potential therapeutic target for the treatment of diseases (gastric cancer) [68]. Remarkably, *GALNT6*, which targets different proteins in cell adhesion and differentiation, was reported to be involved in oncogenic transformation [69]. *CYP4V2*, as a causative gene for Bietti’s Crystalline Dystrophy, belongs to the cytochrome P450 superfamily and encodes fatty acid ω-hydroxylase for both saturated and unsaturated fatty acids [70]. For brain, *CBLN3* was specifically expressed in the cerebellum and may play a critical function as a secreted isoform complex [71]. The cerebrum TSGs (i.e., *NSMF*, *VSTM2L*, and *ENC1*) were mainly involved in the nervous system development. For example, *ENC1,* a member of the ectodermal neural cortex (ENC) gene family, has been reported to regulate neurogenesis [72]. The function of hypothalamus TSGs was related to the physiological activities of viscera organs, including the response to heat and osmotic stress. *Map2k7* can regulate functional plasticity and cognition in the hypothalamus by encoding the JNK activator [73]. The pineal gland TSGs contribute to visual perception (*ROM1* and *UNC119*) [74] and circadian entrainment (*GNB3* and *GNG13*) [75].

Remarkably, several recent studies showed the top tissue-specific genes in the brain (*GRM5*), liver (*SLC22A9*), white blood cell (*FCRL3*), uterus (*TDGF1*), testis (*TRIM69*), lactating mammary gland (*CSN1S1*, *CSN1S2*, *CSN3*, *GLYCAM1*), longissimus muscle, and adipose tissue (*ACACA*, *FASN*, *SCD1*) in cattle [4, 18, 76]. However, these tissue-specific genes were not detected in the current study. This may be explained by the fact that different populations and approaches were used for the identification of tissue-specific genes. In contrast, we identified several TSGs which have been detected in pig, human, and mouse species [77–81], including *MYL3*, *TNNI3*, *MUC5AC*, *MGP*, and *SCGB3A2* (Additional file 15: Table S20). Therefore, we think that these genes are highly conserved across species, which may indicate their potential functions that emerge in particular tissues or organs.

### HUB genes for important biology functions

Co-expression network analysis is an effective strategy to explore the gene connections based on the transcriptional expression. Using WGCNA [82], we divided the filtered 8357 genes into 24 co-expressed gene modules and explored the relationships between 15 modules and tissues based on *r* > 0.65 and *P* < 1.0e−4. It was noted that the cerebellum tissue corresponds to the red module (*r* = 0.73 and *P* = 6.00e−09). The hub genes in the red module (*NSG1*, *CHN1*, *VAMP1*) were mainly involved in the morphological development of neurons. *NSG1* gene was a somatodendritic endosomal membrane protein, which was also highly expressed in neurons [83], and functionally regulates the binding of AMPA receptor and neurotensin receptor [84]. The liver corresponds to the dark green module with 12 core genes (e.g., *SHMT1*, *HSD17B11*, *ETNK2*). As a serine hydroxy-methyltransferase, *SHMT1* was involved in regulating a key reaction in folate-mediated one-carbon metabolism [85]. *ETNK2*, encoding a soluble protein with ethanolamine-specific kinase activity, was found to be highly expressed in the liver in our analysis. The inactivated *ETNK2* can reduce the rate of exogenous ethanolamine synthesis of phosphatidylethanolamine in liver cells [86]. Longissimus dorsi tissue with 18 core genes (e.g., *ENO3*, *TMEM38A*, *TCAP*) was related to the pink module. *ENO3* was regulated by intron muscle-specific enhancers, and this gene can play a parole effect when converting 2-phosphoglycerate into phosphoenolpyruvate in the glycolytic pathway [87]. *TMEM38A* was a specific gene for muscle differentiation, and this gene can be induced in the periphery of the nucleus [88]. As a key regulator of muscle growth, *TCAP* was reported to regulate muscle proliferation and differentiation [89]. Knockout of *TCAP* may damage the normal growth of muscle cells [90]. TFs were involved in gene regulation along with HUBGs identified in co-expression analyses. For example, KLF3 and NFKB1 identified in the pink module (which was enriched in the muscle) may be involved in the growth and development of muscle [91] and skeletal muscle cell differentiation [92]. FOXA2 enriched in a liver acts as a master controller in bile acid homeostasis and bile duct development [93]. HNF4A as a member of the transcriptional regulators of the HNF4 family enriched in the liver was critical for bile acid homeostasis and bile duct development [94].

### Conservative analysis of *HKGs, TSGs, and HUBs* across species

Previous studies suggested that analysis of orthologous genes can enable the exploration of biological functions among species [95–97]. In this study, we performed the comparative analysis of cattle HKGs, TSGs, and HUBs using NCBI HomoloGene [53] database across eight species (see the “Methods” section). We found that fewer ortholog genes (from HKGs, TSGs, and HUBs) were identified in other species with the increase of the evolutionary distance. We also observed that HKGs were more conserved among eight species than TSGs and HUBGs, which confirmed the results of previous studies [47]. In addition, we compared the expression patterns of HKGs, TSGs, and HUBs with other mammals (i.e., human, mouse, rat, and dog). Notably, we found that the expression patterns of HKGs were more conserved among them. Our results indicated that HKGs were mainly involved in regulating gene expression and maintaining basic cell activities, while TSGs and some tissue-specific HUBGs were likely to be generated later during evolution when compared to HKGs, and mainly contributing to tissues-specificity across species [45].

### Tissue-specific expression between beef and dairy cattle

Analysis of the transcriptional difference is an effective strategy to identify breed-specific causal variations related to economic traits (e.g., meat, milk, cashmere) in farm animals [63, 98, 99]. Our study enables us to formulate functional hypotheses on the genetic architecture of tissue-specific gene regulation underlying important traits under distinct selection during breed formation. Notably, we identified several candidate DEGs (e.g., up-regulated genes including *TPM1*, *TPM3*, *COX4I1*, *COX5A*, and *NDUFA4* and down-regulated genes including *SYNM*, *TCAP*, *JPH1*, *DAG1*, *COX1*, and *COX2*) related to muscle differentiation and development between beef and dairy cattle. For instance, TPM encodes an α-helical coiled-coil protein dimer that regulates muscle contraction by the maintenance of thin filament length [100], and this gene was highly expressed in beef cattle as compared to dairy cattle. As members of the TPM gene family, *TPM1* and *TPM3*, regulate the fast and slow fibers in skeletal muscle tissue, respectively [101]. *COX4I1*, a cytochrome c oxidase subunit IV isoform 2, was responsible for regulating the role of oxidative phosphorylation in skeletal muscle adaptation to exercise [102]. The products of *COX5A* and *NDUFA4* regulate the function of the mitochondrial electron transport chain, which plays a vital role in energy metabolism [103]. *SYNM* encodes an intermediate filament protein, which is a typical marker of the smooth muscle cell. The down-regulation of its expression may be functionally related to smooth muscle cell activation in response to vascular injury [104]. As a core driver gene, *TCAP* may affect muscle proliferation and differentiation by regulating myosin [90]. For skeletal muscle, the muscle-specific gene *JPH1* was reported to be involved in the excitatory contraction mechanism and maintaining the stability of newly synthesized JPHs [105]. In addition, we identified several TSGs enriched in pathways related to digestive and metabolic functions in liver tissue. For example, up-regulated DEGs *SCP2* and down-regulated DEGs (*ACOX2* and *HSD3B7*) were linked to a primary bile acid biosynthesis. The highly expressed gene *SCP2* can increase intracellular sterol carrier protein expression and regulate bile acid synthesis [106]. In energy metabolism, several up-regulated genes (*COX7A2*, *COX7B*, *NDUFAB1*, *NDUFB9*, *NDUFC1*, and *SDHA*) and down-regulated genes (*COX2* and *UQCRQ*) were enriched in the oxidative phosphorylation pathway. Cytochrome C oxidase (COX) family members *COX7A2* and *COX7B* regulate the assembly and activation of COX and the activation of mitochondria during oxidative phosphorylation [107]. Two up-regulated genes (*ACADM*, *ACAT1*) and two down-regulated genes (*FADS2*, *HSD17B12*) were enriched in fatty acid metabolism. *ACADM*, encoding a medium-chain acyl-CoA dehydrogenase, plays an essential role in fatty acid oxidation [108].

Moreover, many studies showed that expression levels of *FADS2* were positively correlated with non-alcoholic fatty liver disease, and down-regulated *FADS2* may contribute to the maintaining the activity of delta-6 desaturase to ensure liver digestive function [109]. Overall, the identified DEGs in multiple core tissues between beef and dairy cattle should enable our understanding of their transcriptional regulation on development and functional differentiation of tissue. In addition, our study further supports tissue-specific regulatory patterns which are consistent with the action of natural selection across species, as described in passerine birds [110], primates [111], and high-altitude vertebrates [112], and help to understand the genomic variation and their regulations in diverse breeds that may reflect differences in production traits [4, 27, 113].

## Conclusions

Our study provides the most extensive transcriptome collection of core tissues representing all major organ systems from adult beef cattle to date. A comprehensive analysis of gene expression profiling across tissues can provide necessary information for an in-depth understanding of biological functions. We generated a large-scale gene expression profile across primary tissues in beef cattle, providing valuable information for enhancing genome assembly and annotation. HKGs and TSGs further contribute to better understanding the biology and evolution of multiple tissues in cattle. Identification of DGEs between two breeds (beef vs. dairy purposes) also fills in the knowledge gaps about differential transcriptome regulation of bovine tissues underlying important economic traits.

## Methods

### Bovine tissue samples

Three male Chinese Simmental beef cattle were originated from Hubei Shayang Hanjiang Cattle Development Co., Ltd., Hubei province, China. After weaning, these calves were fattened under the same feeding and management conditions until two years of age. We collected tissue samples under the approval of the Science Research Department of the Institute of Animal Science, Chinese Academy of Agricultural Sciences under protocol IAS2020-33. A total of 135 tissue samples from the major organ systems (e.g., the digestive system, immune system) were divided into 51 tissue types in biological replicates. Samples were saved by RNAlater (Qiagen) and snap-frozen in liquid nitrogen. Details of samples were included in Additional file 1: Table S1. We retrieved the dataset of dairy cattle tissue samples from the publication [4].

### Library preparation and sequencing

Total RNA was extracted from 135 tissues samples using Trizol method, and RNA was subjected to quality control by the NanoDrop® 2000 (Thermo, CA, USA) and treated with DNase I (RNase-free) following the manufacturer’s instructions. RNA purity and integrity were assessed by agarose gel electrophoresis. RNA concentration was measured using a Qubit RNA BR Assay Kit (Q10210; Thermo Fisher Scientific, Carlsbad, CA, USA) and RNA integrity was detected by the Agilent Bioanalyzer 2100 system (Agilent Technologies, Santa Clara, CA, USA). A total amount of 5 μg RNA per sample was used for RNA-seq library preparation. mRNA libraries were generated by Novogene Co., Ltd. using the Illumina TruSeq Stranded library protocols and sequenced on the Illumina HiSeq 2500 sequencing platform (Illumina, San Diego, CA, USA). Briefly, Poly-(A) mRNA was isolated from total RNA using Oligo-(dT) magnetic beads and then fragmented in fragmentation buffer. First-strand cDNA was synthesized using a six-base random primer. Double-stranded (ds) cDNA was then synthesized using buffer solution, dNTPs, RNaseH, DNA polymerase I, and RNase H. The library fragments were purified with AMPure XP beads (Beckman Coulter, Beverly, MA, USA) to generate cDNA fragments preferentially 250–300 bp in length. To ensure the quality of the library, PCR products were purified (AMPure XP beads) and quality was assessed on the Agilent Bioanalyzer 2100 system. The cBot Cluster Generation System using TruSeq PE Cluster Kit v4-cBot-HS (Illumina) was used to cluster the index-coded samples. Then the unform library preparations were sequenced on an Illumina Hiseq-PE150 platform.

### Transcriptional sequencing and processing

The raw data (short reads) of transcriptional sequencing for the bovine tissues were processed with FASTP (0.21.0) [114], HISAT2 (v.2.1.0) [42] and STRINGTIE (v.2.1.1) [115]. Clean reads were obtained by removing reads containing adapter, reads containing ploy-N (*N*≥10%), and low-quality reads (*Q*≤5) using FASTP software with default parameters [114]. The GC content of the clean data was assessed. The clean data with high quality was used in all following analyses. The index of the ARS-UCD1.2 reference genome was built using the HISAT2-build software. Clean data for each sample were mapped to the reference genome using HISAT2. Samtools (v.1.9) was used to sort and convert the SAM files to BAM format [116]. The transcripts and genes for each sample were assembled and quantified by STRINGTIE (v.2.1.1). After assembling each dataset, all transcripts were merged into a unique transcriptome by STRINGTIE’s merge function, including all transcripts included in the annotation and the novel transcripts. The transcript abundances and gene expression levels were estimated based on read counts and fragments per kilobase of transcript per million mapped reads (FPKM), respectively. Finally, the GFFCOMPARE v.1 utility was used to compare predicted transcripts produced by STRINGTIE with the ARS-UCD1.2.

### Gene expression pattern across tissues

To investigate gene expression patterns of 51 bovine tissue types, we first retained the gene expression in at least two replicate samples and then calculated the mean of gene expression values in replicate samples (two replicate samples of 12 tissues and three replicate samples of 36 tissues). The expression level of each gene was converted using log-transformation (log_2_ (FPKM+1)), and the tissues clusters were visualized using PCA. In addition, hierarchical clustering was generated using Pheatmap package (v.1.0.12).

### Housing-keeping genes categorization

We strictly defined HKGs as genes showing constitutive expression in all most tissues [45]. To study the expression changes of each HKG in the bovine expression profile, we used the CV to evaluate the degree of variation for each gene [66]. In brief, CV was defined as the ratio of the standard deviation to the mean. The calculation formula was as follows. CV = σ/μ, where CV represents the variation degree for genes between tissues; σ represents the standard deviation; μ represents the mean. We used the quartile of the total distribution of CV values to divide the HKGs into lowly variable expression (CV ≤ first quartile), mediumly variable expression (first quartile < CV < third quartile), and highly variable expression (CV ≥ third quartile). To explore the functional differences of HKGs with low, medium, and high expression variability, we performed GO enrichment analysis on HKGs [55]. We defined constantly expression genes within lowly variable expression HKGs, whose FPKM value fulfills the log_2_ (FPKMmax/FPKMmin) < 2. Moreover, HKGs with average expression FPKM > 50 in all bovine tissues were determined as candidate reference HKGs. The hierarchical clustering map of HKGs was drawn using the Pheatmap package based on Pearson’s correlation coefficient.

### Tissue-specific gene detection

For the detection of TSGs, we used more stringent standards as previously described [47]. In brief, TSGs were defined that the gene with expression level (FPKM) in the tissue according to criteria: (1) the FPKM value of the candidate gene in one tissue was more than three times that of other tissue, (2) the FPKM value of candidate gene target tissue was greater than 50% of the average expression level in all other tissues, and (3) the expression level of candidate genes was at the top 25% of all genes in each tissue. We classified 51 bovine tissues into 10 organ systems based on biological categories as described by Harhay et al. [18]. Functional annotation and enrichment analysis were based on KOBAS 2.0 [117]. The Pheatmap package was used to calculate Pearson’s correlation coefficient and generate a hierarchical clustering map for 477 TSGs identified in 51 bovine tissue types. In addition, the protein interaction network of TSGs in different organ systems was generated using the STRING database [48], and the network was visualized using Cytoscape software (v.3.7.1) [118].

### Gene network cluster analysis and construction

The co-expression network analysis of the gene expression was performed using a weighted gene co-expression network analysis (WGCNA) package (v.1.69) in R programming [82]. Briefly, we first constructed an expression matrix of 8,357 genes (FPKM > 1) using 51 bovine tissues. The cluster analysis on all tissues uses the *hclust* function with the average agglomeration method. The soft threshold (β) was determined based on scale-free distribution. The step-by-step and the dynamic cutting methods were used to construct the gene network and module detection. The parameters were the minimum module size of 30 and mergeCutHeight 0.25. PCA was performed on the expression matrix of genes in each module to obtain module eigengene (ME). To investigate the relationship between the modules and tissues, we constructed a design matrix X, and each row and each column corresponded to a sample (the diagonal was 1, the non-diagonal was 0). The correlation coefficient between the matrix X and the module ME was calculated using the Pearson correlation coefficient. The module with a correlation coefficient larger than 0.65 was considered as the tissue-specific module. Moreover, functional enrichment analyses for tissue-specific module genes were performed using DAVID with FDR multiple correction [55]. The network connectivity (kTotal) and the connectivity within the module (kWithin) were estimated, and genes at the top 5% of kWithin were selected as the HUBGs. The HUBG network in the tissue-specific module was constructed using Cytoscape (v.3.7.1). Moreover, co-expressed genes in the most correlated modules for liver and muscle tissues were used to predict TFs using iRegulon tool (v.1.3) [119], and the results of TFs prediction were obtained from the 20kb upstream of the transcription start site. The regulation network of TFs involved with co-expressed genes was constructed using Cytoscape (v.3.7.1). Normalized enrichment scores (NES) were used as estimators of regulation relative activity.

### Conservation model and expression analysis across species

To investigate functional conservation of identified genes in bovine atlas across species, we explored orthologs of bovine genes in other eukaryotic using NCBI homologous database [53]. We mapped the full gene sets, HKGs, TSGs, and HUBGs in the bovine gene expression atlas to humans, mice, rats, dogs, chickens, fly (*D. melanogaster*), worms (*C. elegans*), and yeast (*S. cerevisiae*) species to obtain orthologous genes. Student’s *t* test was applied to estimate the correlation between the orthologous genes of HKGs, TSGs, HUBGs, and full gene sets.

### Differential gene expression patterns between beef and dairy cattle

To evaluate differential expression patterns between beef and dairy cattle, we retrieved the dairy cattle data, including brain, heart, liver, lung, muscle, and testis, from a previous publication [4]. To adjust the batch effect of mRNA data across studies, we considered genes with FPKM larger than one in three biological replicates as candidate genes and identified the orthologous genes of beef and dairy cattle based on the Ensemble database (http://asia.ensembl.org/). We used the TMM (trimmed mean of M values) normalization implemented in the edgeR package [120] to eliminate the difference between sequencing libraries. Scale factors were used to measure the differences between tissues and breeds. To identify DEGs between beef and dairy cattle, we considered genes with an absolute log_2_ (fold change) (log_2_FC) > 1 and False Discovery Rate (FDR) adjusted *P* value < 0.005 as candidates. Intersection sets of DEGs between six tissues were visualized using UpSetR package [121]. The Pheatmap package (v.1.0.12) was used to calculate the correlation coefficient and construct tissue clustering based on the DEGs. We finally performed a functional enrichment analysis of DEGs using DAVID [55].

### Real-time quantitative PCR (RT-qPCR) analysis of TSGs

To validate TSGs, we randomly selected 12 TSGs for RT-qPCR analyses with QuantStudio 7 Flex real-time PCR System (Life Technologies, Carlsbad, CA, USA). Total RNA from muscle, liver, brain, and abomasum samples were extracted using Trizol method. The cDNA was synthesized by reverse TSGs using Prime Script™ RT Reagent kit with gDNA Eraser (Takara, Dalian, China). RT-qPCR primers for all genes were designed using the Primer Premier 5 and synthesized by Sangon Biotech (Sangon, Shanghai, China). The 10-μL RT-qPCR reaction contained 0.5 μL cDNA, 0.4 μL of each primer (F/R), 5 μL 2 × KAPA SYBR® FAST (KAPABiosystems, Wilmington, MA, USA), 0.2 μL Rox Low, 3.5 μL H_2_O. The amplification cycle was as follows: initial denaturation for 3 min at 95 °C for 1 cycle, followed by 40 cycles at 95 °C for 2 s and 60 °C for 20 s. The 2^−ΔΔCt^ method was used to transform Ct values, and the results were estimated by Student’s *t* test. For validation, three samples were used for qPCR experiments, and each sample had three technical replicates. The *GAPDH* was used as an endogenous control to normalize gene expression levels.

## Supplementary Information

**Additional file 1: Table S1.** Sample information and classification of tissue samples from beef cattle in this study. **Table S2.** The summary of raw reads, clean reads and the total mapped read rate. **Table S3.** Assemble the transcript statistics of each sample with the merged transcripts file. **Table S4.** Comparing the transcript assembly with reference annotation. **Table S5.** The relationship between the predicted transcript and reference annotated files.

**Additional file 2: Figure S1.** The coefficient of variation (CV) distribution of the gene expression profile and HKGs.

**Additional file 3: Table S6.** The expression levels of 32 HKGs across 51 tissues.

**Additional file 4: Figure S2.** Expression of 32 HKGs across 51 tissue types. **Figure S3.** Expression level of 8 commonly housekeeping genes across 51 tissue types.

**Additional file 5: Table S7.** Tissue specific genes (TSGs) and tissue-specific transcripts (TSTs) identified based on stringent criteria.

**Additional file 6: Figure S4.** Expression pattern of several top TSG in all other system. **Figure S5.** GO function enrichment analysis in other system category.

**Additional file 7: Table S8.** Tissue specific gene (TSG) function annotation (Top5) and pathway enrichment (Top5).

**Additional file 8: Figure S6.** Clustering of expression patterns of 48 TSGs in brain. **Figure S7.** Hierarchical clustering of 51 tissues of cattle. **Figure S8.** The determination of the power Beta (β) value is based on the scale free topology criterion and mean connectivity under the weighted gene correlation network analysis (WGCNA) method. **Figure S9.** Network scale-free topology distribution test based on selected β value.

**Additional file 9: Table S9.** The identified functional modules using dynamic cutting method. **Table S10.** Tissue-specific modules detection and hub genes screening. **Table S11.** Tissue-specific module gene function annotation (Top10). **Table S12.** The intramodular connectivity for con-expression analysis calculated for each gene. **Table S13.** The identified Hub gene in tissue-specific module (Top5%).

**Additional file 10: Figure S10.** TF analysis of liver and muscle tissue specificity correlated module.

**Additional file 11: Table S14.** Tissue-specific module transcription factor analysis. **Table S15.** Cross-species conservation of HKGs, TSGs, HUBGs and full genes set. **Table S16.** The expression level of cattle full genes set, HKGs, TSGs and HUBGs in mammals. **Table S17.** Identification of DEGs between beef and dairy cattle. **Table S18.** Functional annotation (for Top10) and pathway enrichment (for Top10) analysis of DEGs identified in six tissues between beef and dairy cattle.

**Additional file 12: Figure S11.** Pathway enrichment of DEGs in muscle tissue between beef and dairy cattle.

**Additional file 13: Table S19.** RT-qPCR primer sequences of TSGs in the current study.

**Additional file 14: Figure S12.** Validation of the expression levels of beef cattle TSGs using RT-qPCR. **Figure S13.** Technical validation of RNA-seq results using RT-qPCR by correlation analysis.

**Additional file 15: Table S20.** Summary of shared TSG identified in this study and previous studies.

**Additional file 16.**

## Data Availability

All beef cattle RNA-Seq data generated by this study have been submitted to the National Genomics Data Center (NGDC) (https://bigd.big.ac.cn/) under accession numbers PRJCA004358 [122]. All dairy cattle RNA-Seq data in this study were downloaded from the NCBI Gene Expression Omnibus (GEO; https://www.ncbi.nlm.nih.gov/geo/) under accession numbers GSE128075 [123].

## Abbreviations

HKGs: Housekeeping genes
TSGs: Tissue-specific genes
HUBGs: HUB genes
TSTs: Tissue-specific transcripts
DEGs: Differentially expressed genes
CV: Coefficient of variation
FPKM: Fragments per kilobase of transcript per million mapped reads
WGCNA: Weighted gene co-expression network analysis
TF: Transcription factor
MF: Molecular function
BP: Biological process
CC: Cellular component
PCA: Principal component analysis
ME: Module eigengene
TMM: Trimmed mean of M values
