# Supplementary material for: Transcriptional atlas analysis from multiple tissues reveals the expression specificity patterns in beef cattle
Source: BMC Biol. 2022 Mar 29;20:79. doi: 10.1186/s12915-022-01269-4 (PMC8966188; doi:10.1186/s12915-022-01269-4)
Supplement: Supplementary file 2 — Additional file 2: Figure S1. The coefficient of variation (CV) distribution of the gene expression profile and HKGs. [file 12915_2022_1269_MOESM2_ESM.docx]

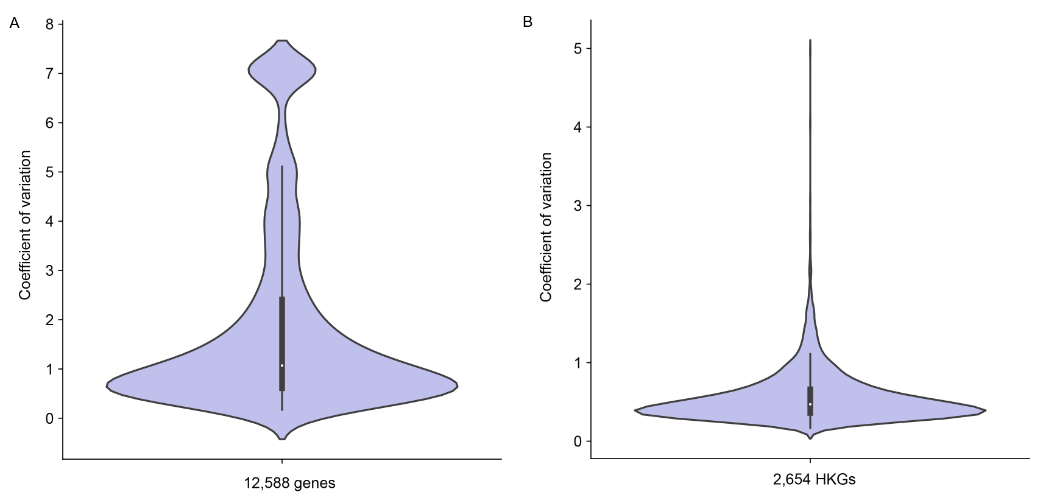


**Figure. S1**. **The coefficient of variation (CV) distribution of the gene expression profile and HKGs**. **a**. The CV distribution of 12,588 gene sets. **b**. The CV distribution of 2654 HKGs.
