# Supplementary material for: Transcriptional atlas analysis from multiple tissues reveals the expression specificity patterns in beef cattle
Source: BMC Biol. 2022 Mar 29;20:79. doi: 10.1186/s12915-022-01269-4 (PMC8966188; doi:10.1186/s12915-022-01269-4)
Supplement: Supplementary file 4 — Additional file 4: Figure S2. Expression of 32 HKGs across 51 tissue types. Figure S3. Expression level of 8 commonly housekeeping genes across 51 tissue types. [file 12915_2022_1269_MOESM4_ESM.docx]

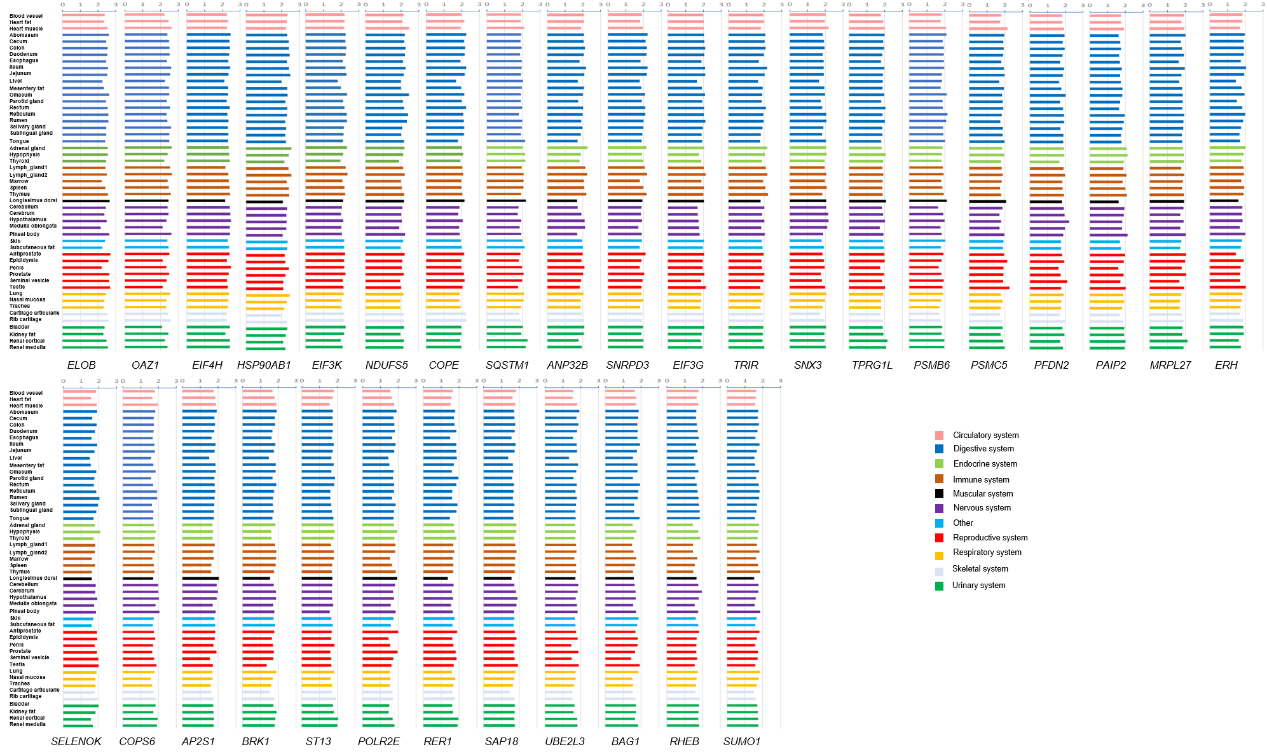


**Figure. S2**. **Expression of 32 HKGs across 51 tissue types**. The expression levels of HKGs were normalized by log_10_. The length of the horizontal bar represents the intensity of gene expression in the tissue. The colors of the horizontal bars represent different system categories.


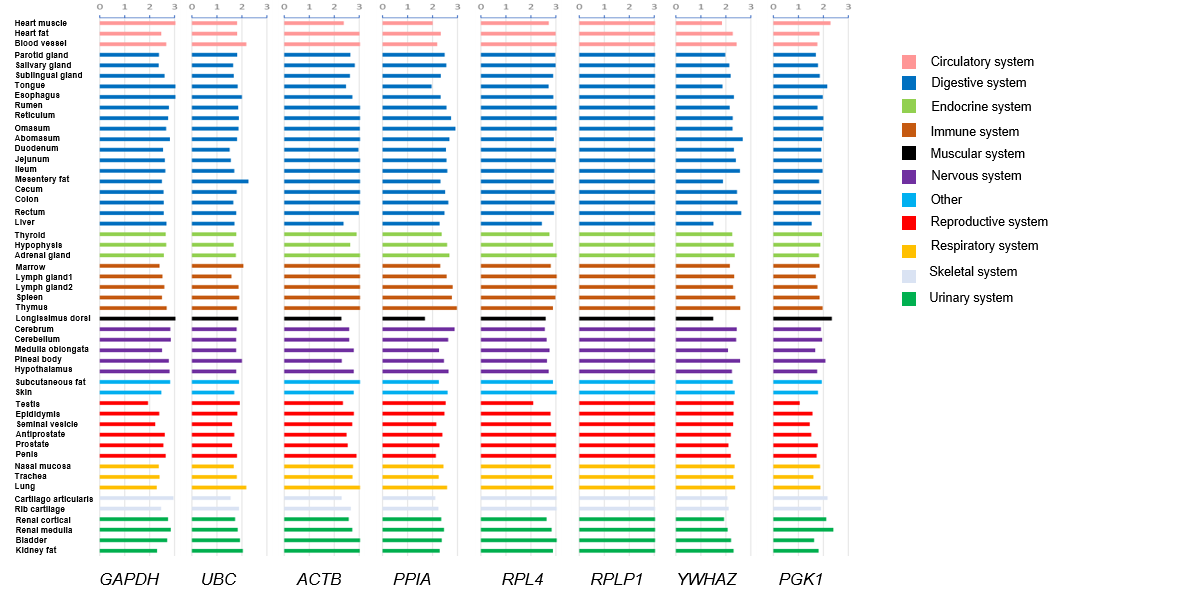


**Figure. S3.** **Expression level of 8 commonly housekeeping genes across 51 tissue types**. The expression levels of HKGs were normalized by log_10_. The length of the horizontal bar represents the intensity of gene expression in the tissue. The colors of the horizontal bars represent different system categories based on known biological.
