# Supplementary material for: Transcriptional atlas analysis from multiple tissues reveals the expression specificity patterns in beef cattle
Source: BMC Biol. 2022 Mar 29;20:79. doi: 10.1186/s12915-022-01269-4 (PMC8966188; doi:10.1186/s12915-022-01269-4)
Supplement: Supplementary file 6 — Additional file 6: Figure S4. Expression pattern of several top TSG in all other system. Figure S5. GO function enrichment analysis in other system category. [file 12915_2022_1269_MOESM6_ESM.docx]

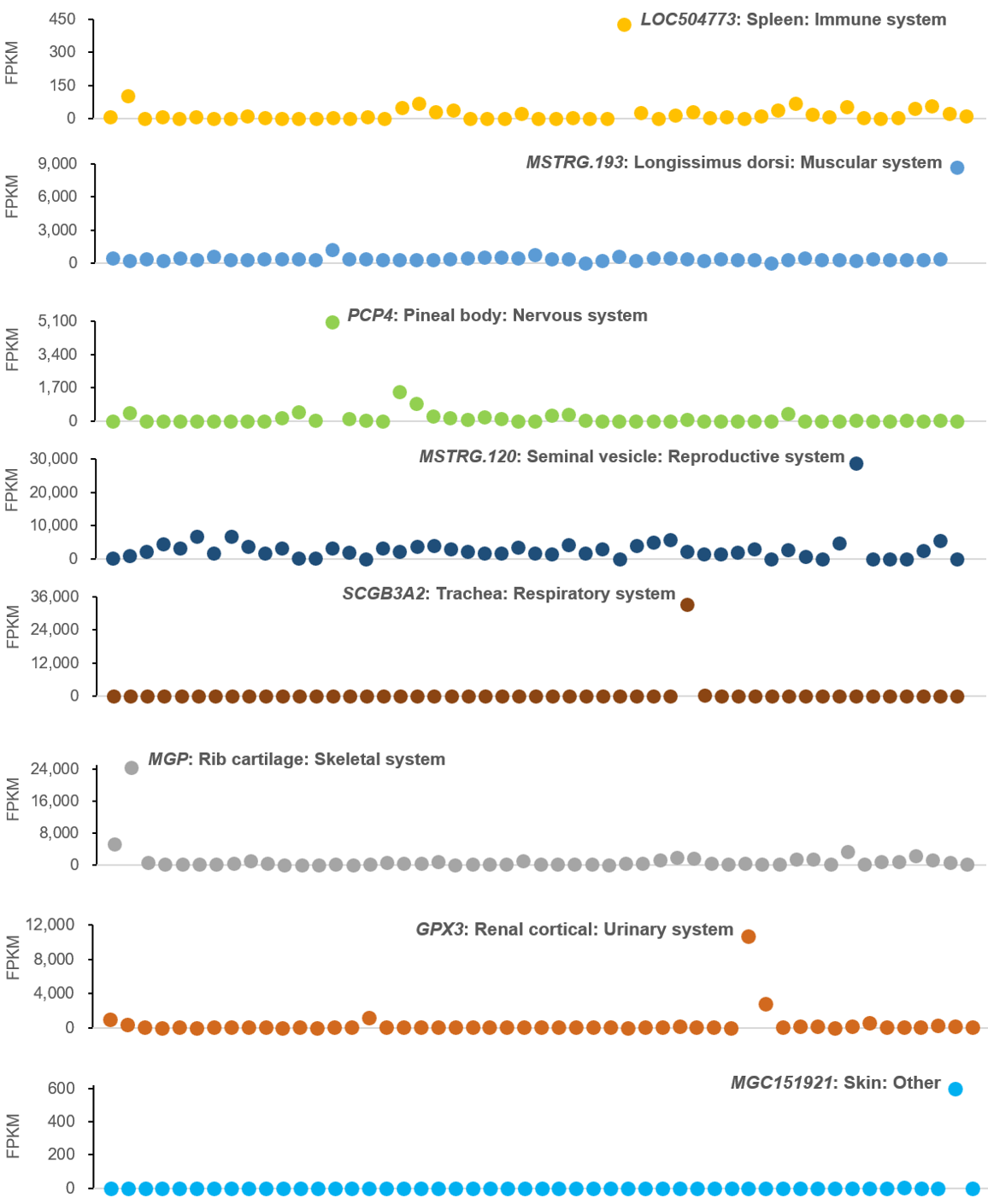


**Figure S4.** **Expression pattern of several top TSG in all other system**. Top TSG includes *LOC504773* (in the immune system), *MSTRG.193* (in the muscular system), *PCP4* (in the nervous system), *MSTRG.120* (in the reproductive system), *SCGB3A2* (in the respiratory system), *MGP* (in the skeletal system), *GPX3* (in the urinary system) and *MGC151921* (in others). The y-axis is the raw gene expression (FPKM), x-axis represents tissue.


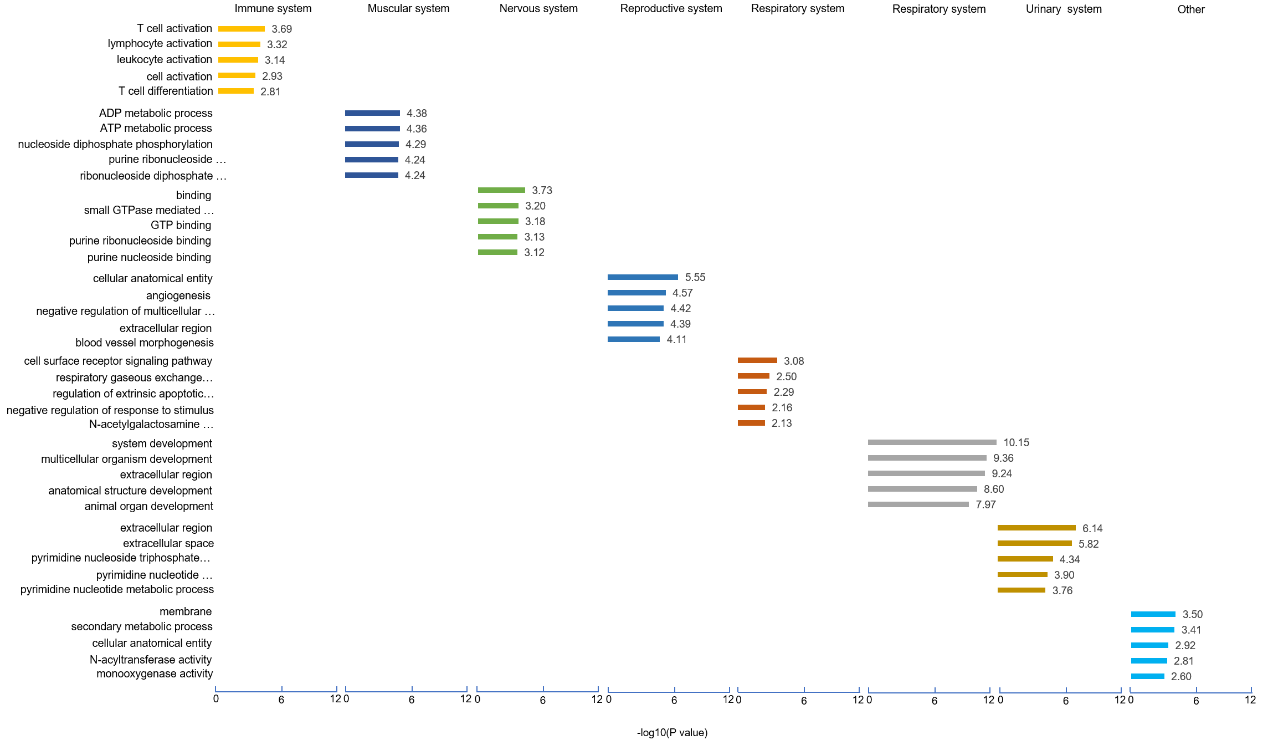


**Figure. S5. GO function enrichment analysis in all other system category**. The x-axis represents –log_10_(P value), the y-axis represents GO term.
