# Supplementary material for: Transcriptional atlas analysis from multiple tissues reveals the expression specificity patterns in beef cattle
Source: BMC Biol. 2022 Mar 29;20:79. doi: 10.1186/s12915-022-01269-4 (PMC8966188; doi:10.1186/s12915-022-01269-4)
Supplement: Supplementary file 8 — Additional file 8: Figure S6. Clustering of expression patterns of 48 TSGs in brain. Figure S7. Hierarchical clustering of 51 tissues of cattle. Figure S8. The determination of the power Beta (β) value is based on the scale free topology criterion and mean connectivity under the weighted gene correlation network analysis (WGCNA) method. Figure S9. Network scale-free topology distribution test based on selected β value. [file 12915_2022_1269_MOESM8_ESM.docx]

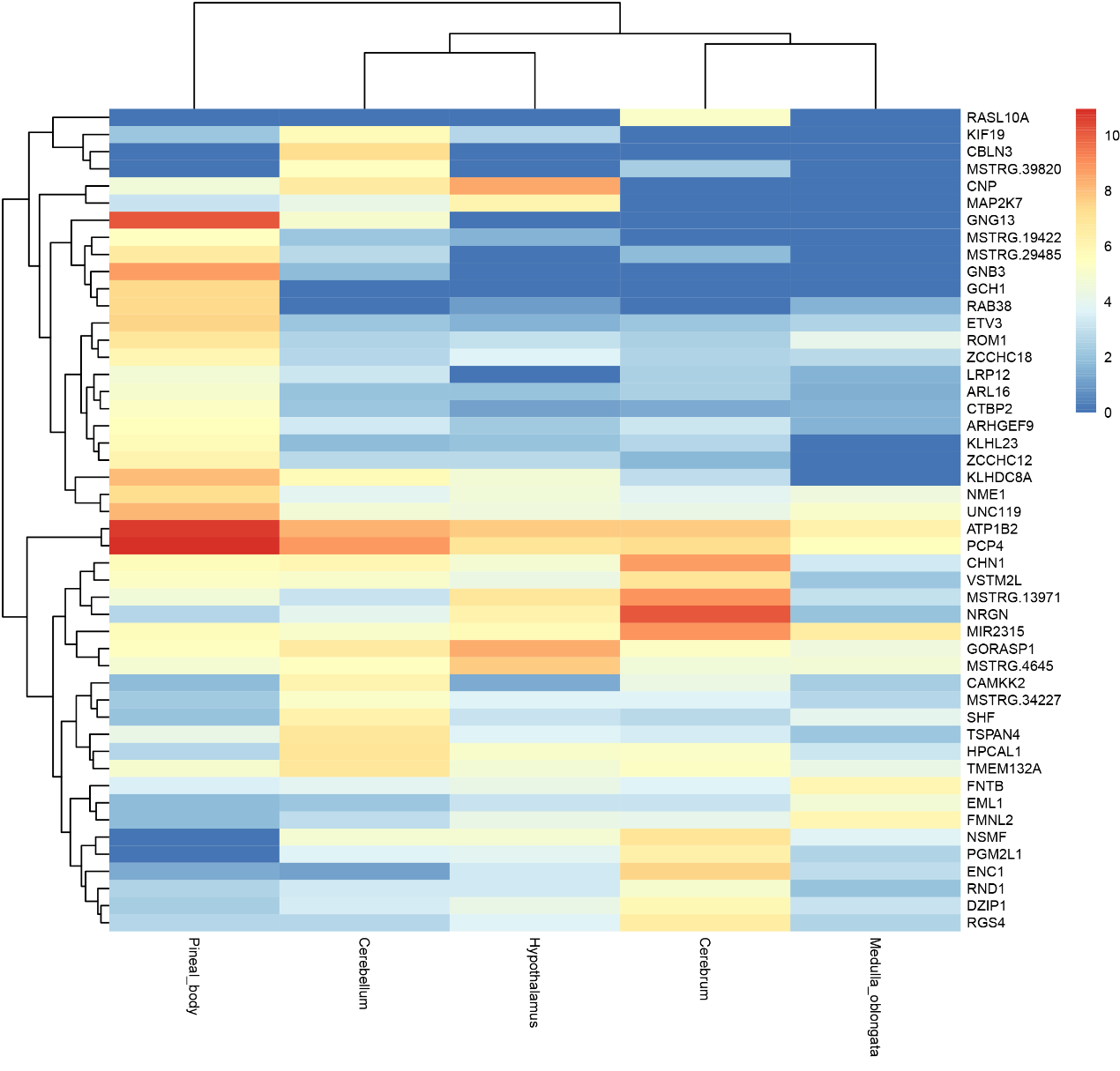


**Figure S6**. **Clustering of expression patterns of 48 TSGs in brain**. Color intensity represents expression level estimated through log_10_ normalized FPKM, red represents highly-expressed and blue represents lowly-expressed. The x-axis represents tissues, namely the pineal gland, cerebellum, hypothalamus, cerebrum and medulla oblongata, the y-axis represents TSGs expression levels.


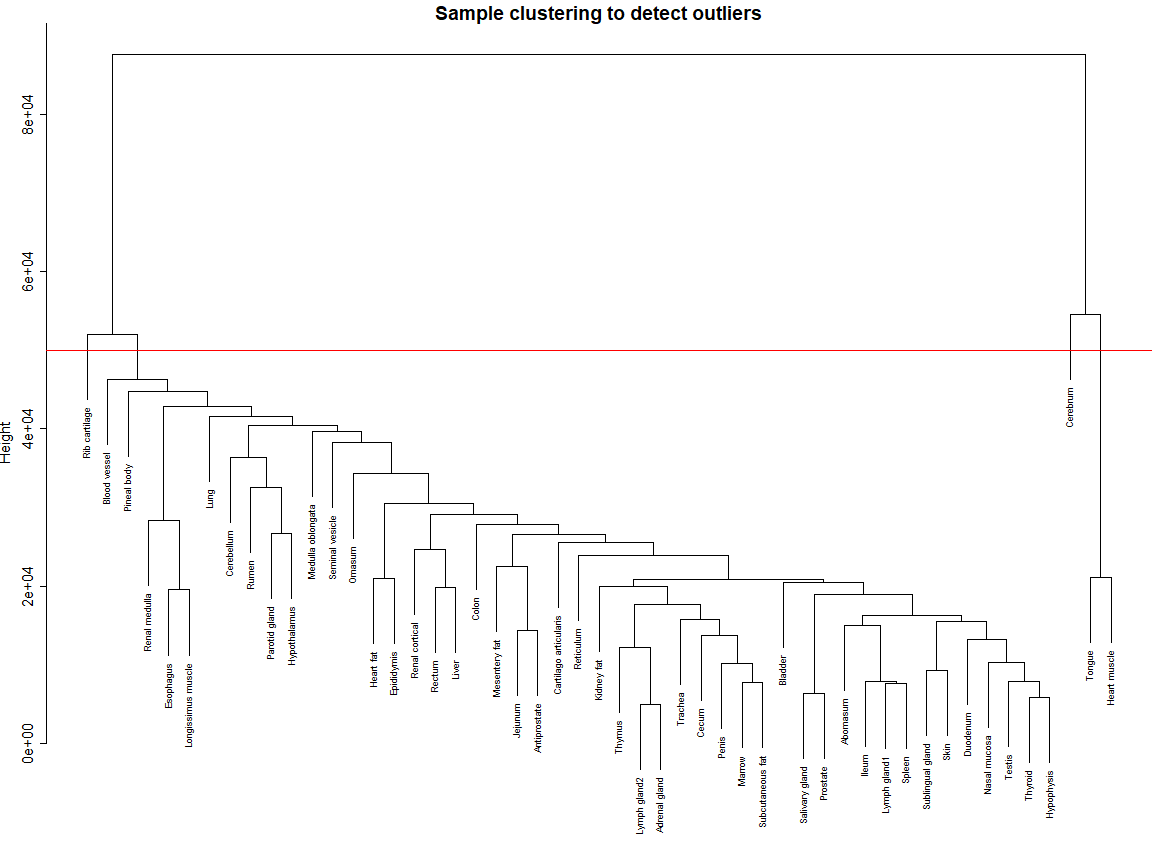


**Figure. S7. Hierarchical clustering of 51 tissues of cattle**.


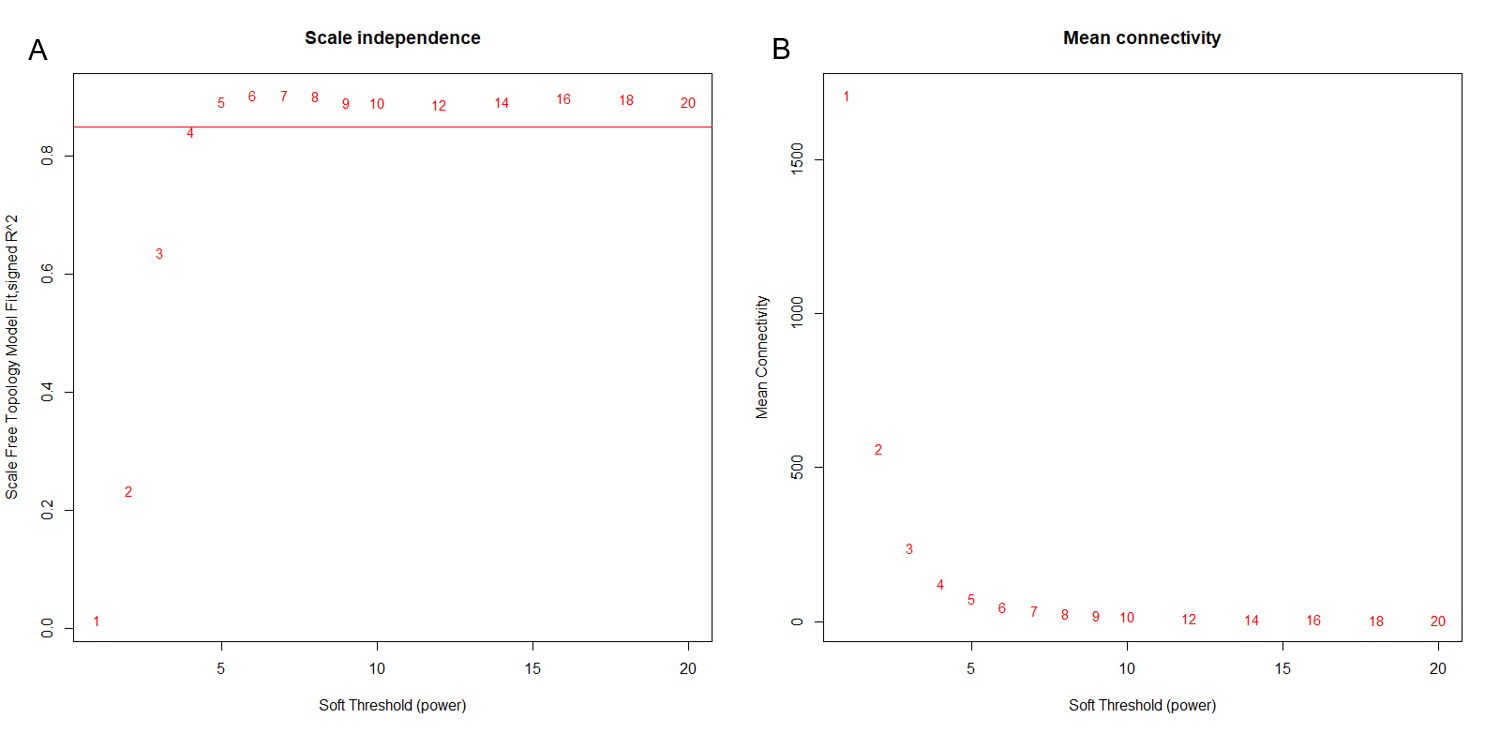


**Figure S8**. **The determination of the power Beta (β) value is based on the scale free topology criterion and mean connectivity under the weighted gene correlation network analysis (WGCNA) method**. To ensure that the average connectivity of the network is smooth, we chose β=4 based on both chart: **(a)** topology fitting results and **(b)** mean connectivity.


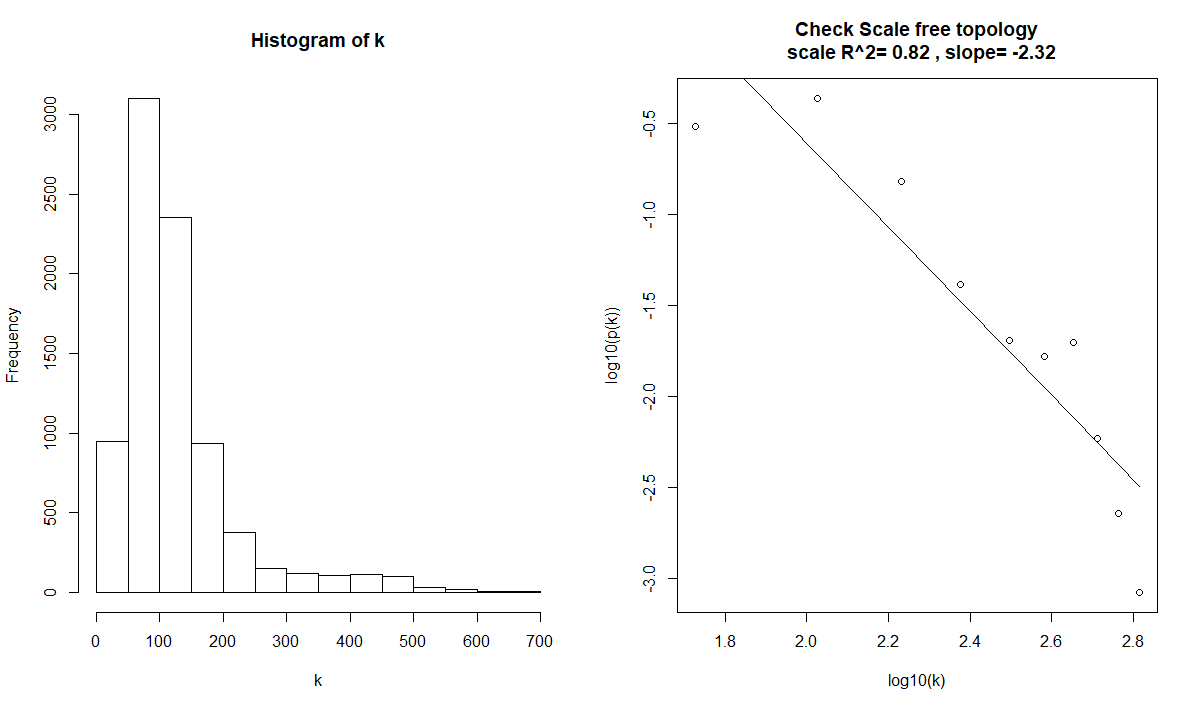


**Figure. S9. Network scale-free topology distribution test based on selected β value**.
