# Supplementary material for: Transcriptional atlas analysis from multiple tissues reveals the expression specificity patterns in beef cattle
Source: BMC Biol. 2022 Mar 29;20:79. doi: 10.1186/s12915-022-01269-4 (PMC8966188; doi:10.1186/s12915-022-01269-4)
Supplement: Supplementary file 10 — Additional file 10: Figure S10. TF analysis of liver and muscle tissue specificity correlated module. [file 12915_2022_1269_MOESM10_ESM.docx]

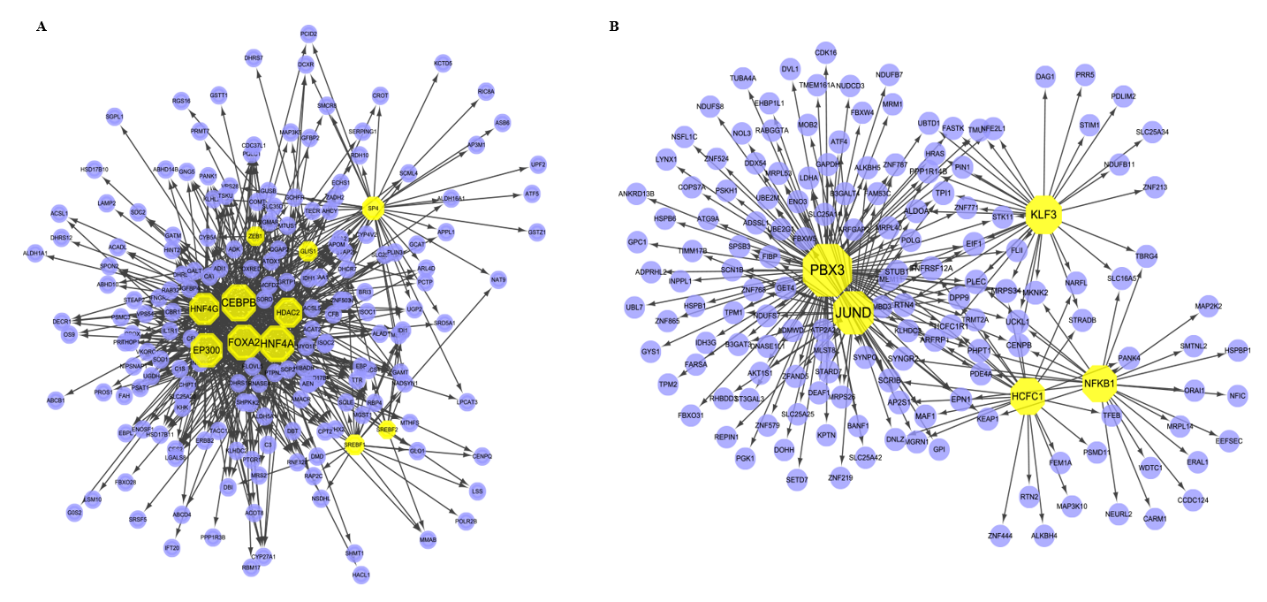


**Figure S10**. **TF analysis of liver and muscle tissue specificity correlated module**. **a**. TF analysis of liver-darkgreen module genes. Yellow nodes represent transcription factors and blue nodes represent regulatory target genes. **b**. TF analysis of muscle-pink module genes. Yellow nodes represent transcription factors and blue nodes represent regulatory target genes.
