# Supplementary material for: Transcriptional atlas analysis from multiple tissues reveals the expression specificity patterns in beef cattle
Source: BMC Biol. 2022 Mar 29;20:79. doi: 10.1186/s12915-022-01269-4 (PMC8966188; doi:10.1186/s12915-022-01269-4)
Supplement: Supplementary file 12 — Additional file 12: Figure S11. Pathway enrichment of DEGs in muscle tissue between beef and dairy cattle. [file 12915_2022_1269_MOESM12_ESM.docx]

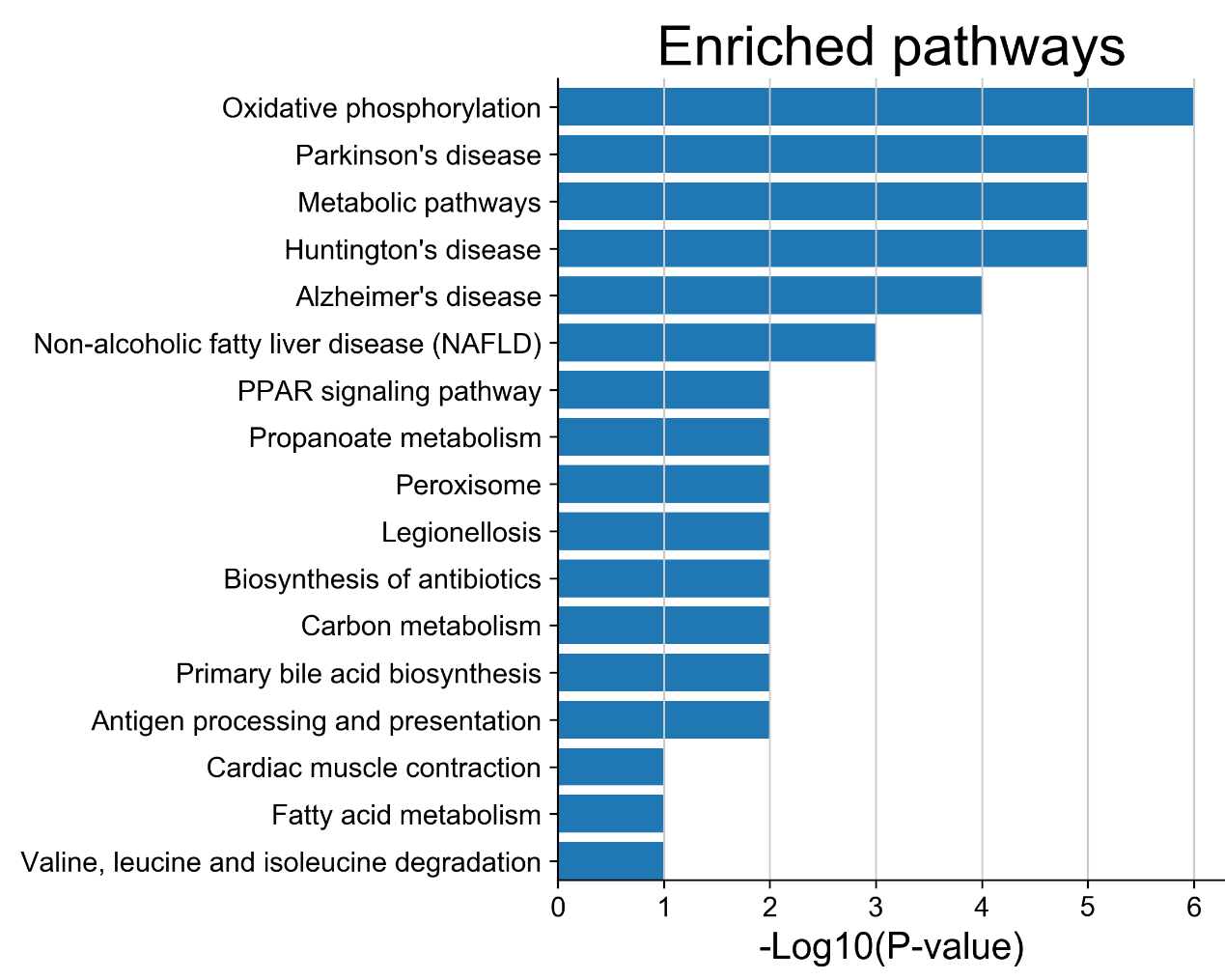


**Figure S11**. **Pathway enrichment of DEGs in muscle tissue between beef and dairy cattle**. The x-axis represents the significance level of the pathway, expressed as -log_10_ (P-value), and the y-axis represents the pathway term.
