# Supplementary material for: Transcriptional atlas analysis from multiple tissues reveals the expression specificity patterns in beef cattle
Source: BMC Biol. 2022 Mar 29;20:79. doi: 10.1186/s12915-022-01269-4 (PMC8966188; doi:10.1186/s12915-022-01269-4)
Supplement: Supplementary file 14 — Additional file 14: Figure S12. Validation of the expression levels of beef cattle TSGs using RT-qPCR. Figure S13. Technical validation of RNA-seq results using RT-qPCR by correlation analysis. [file 12915_2022_1269_MOESM14_ESM.docx]

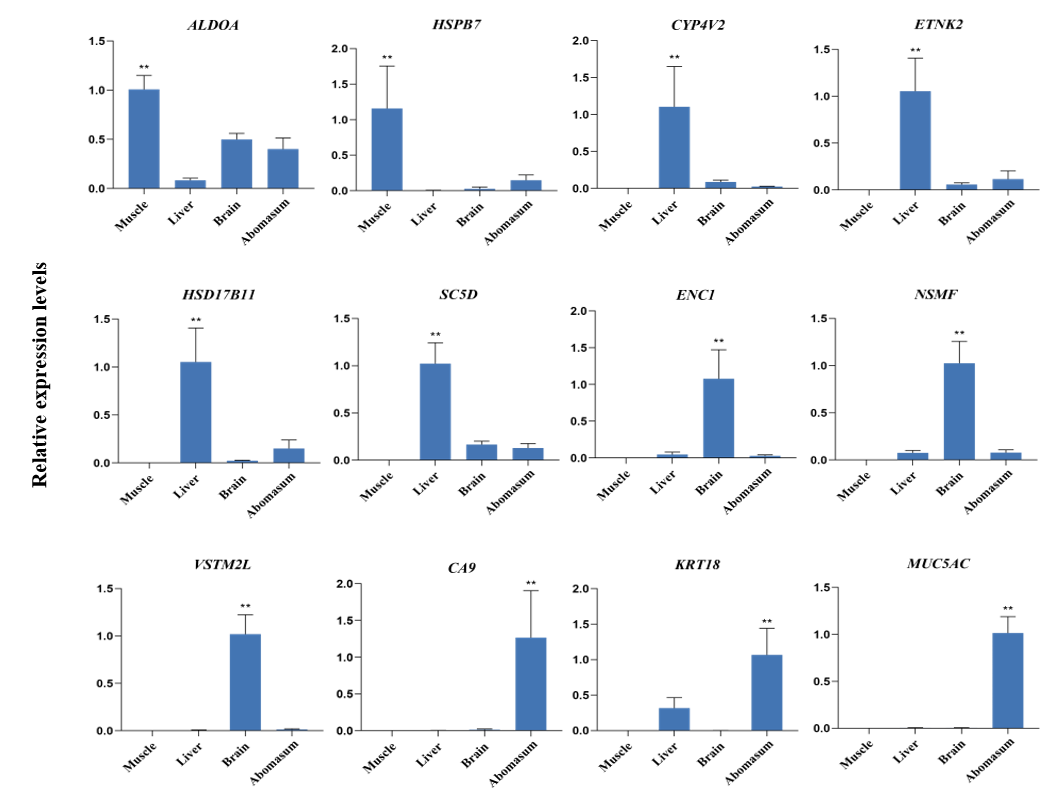


**Figure S12**. **Validation of the expression levels of beef cattle TSGs using RT-qPCR**. Histograms show mRNA expression levels of twelve TSGs randomly selected in tissues (including muscle, liver, brain and abomasum) of Chinese Simmental beef cattle (n = 3). Results represent Mean ± SEM. ** P < 0.01.


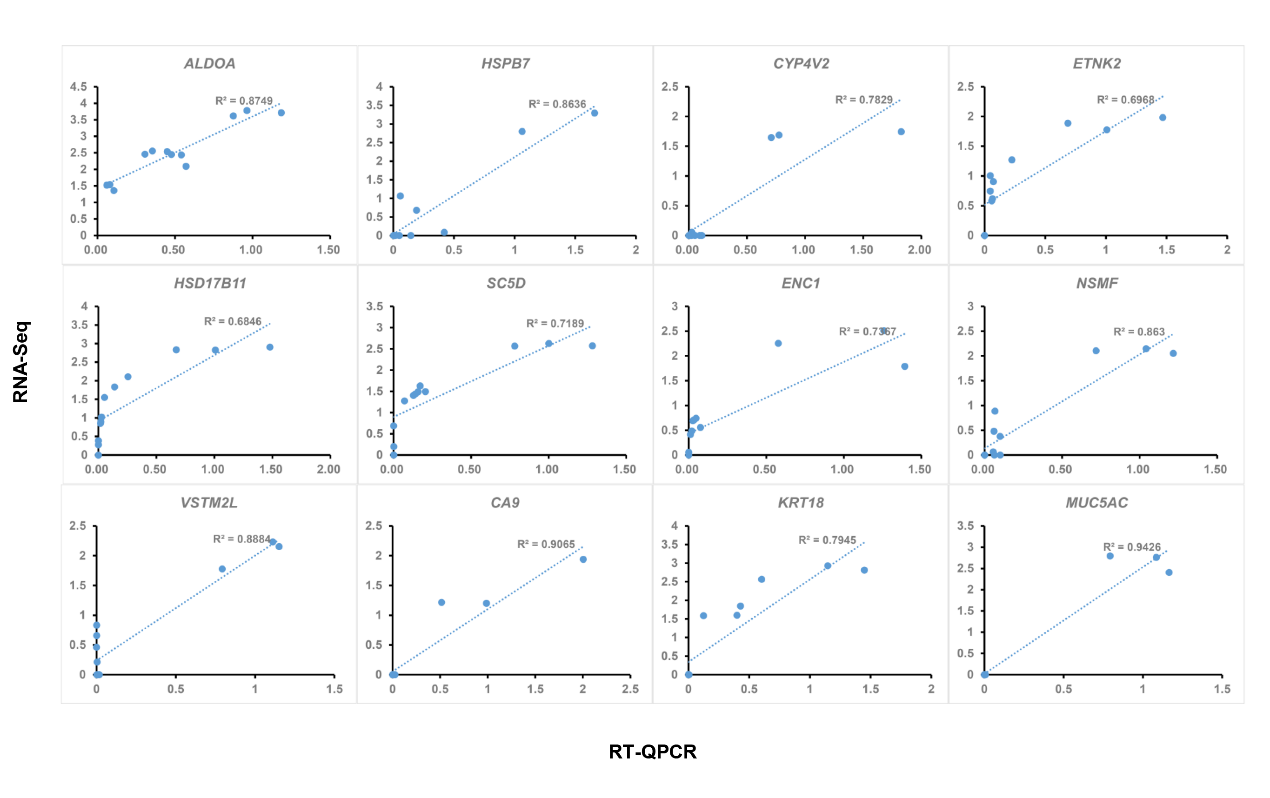


**Figure S13**. **Technical validation of RNA-seq results using RT-qPCR by correlation analysis**. The RT-qPCR value was calculated by 2^-ΔΔCT^. The FPKM value of the above gene were normalized using log_10_ (FPKM) in RNA-seq.
