# Supplementary figures and images for: Transcriptional atlas analysis from multiple tissues reveals the expression specificity patterns in beef cattle
Source: BMC Biol. 2022 Mar 29;20:79. doi: 10.1186/s12915-022-01269-4 (PMC8966188; doi:10.1186/s12915-022-01269-4)

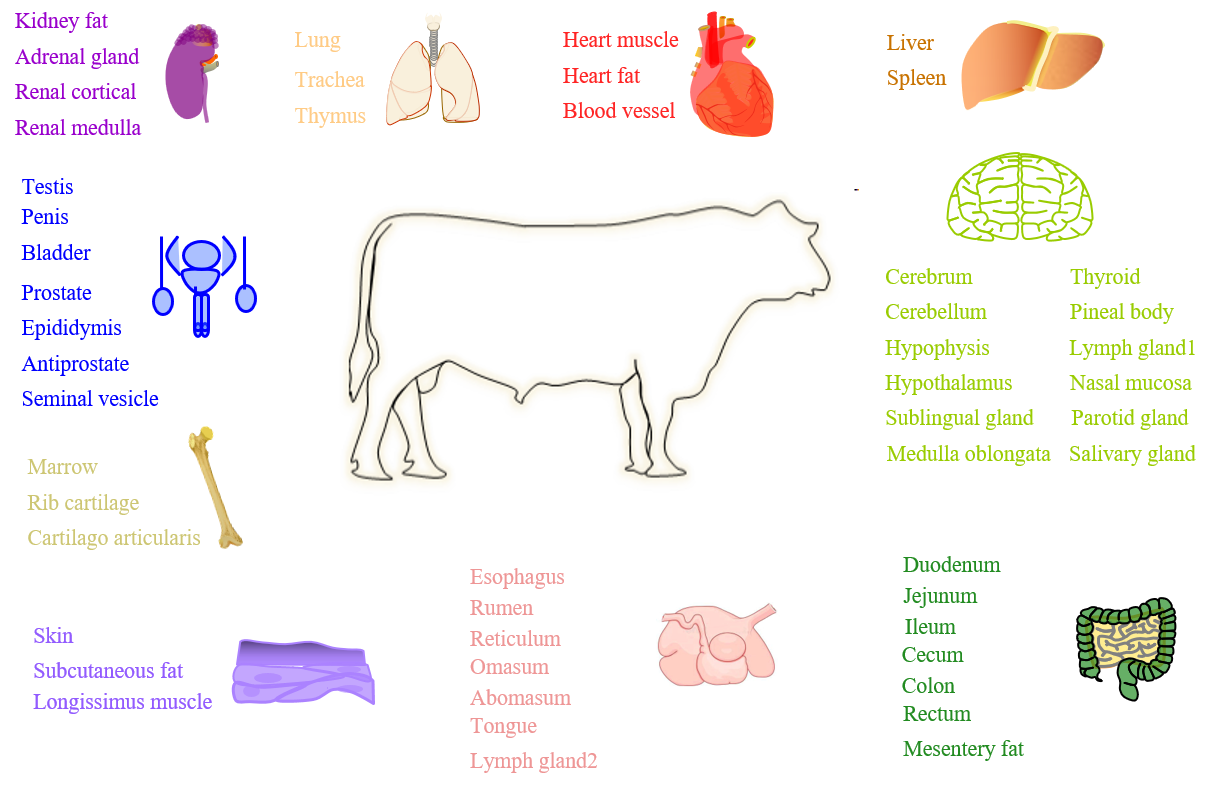

Supplement: Supplementary file 16 — Additional file 16. [file 12915_2022_1269_MOESM16_ESM.png]
